# Splicing misregulation of *SCN5A* contributes to cardiac-conduction delay and heart arrhythmia in myotonic dystrophy

**DOI:** 10.1038/ncomms11067

**Published:** 2016-04-11

**Authors:** Fernande Freyermuth, Frédérique Rau, Yosuke Kokunai, Thomas Linke, Chantal Sellier, Masayuki Nakamori, Yoshihiro Kino, Ludovic Arandel, Arnaud Jollet, Christelle Thibault, Muriel Philipps, Serge Vicaire, Bernard Jost, Bjarne Udd, John W. Day, Denis Duboc, Karim Wahbi, Tsuyoshi Matsumura, Harutoshi Fujimura, Hideki Mochizuki, François Deryckere, Takashi Kimura, Nobuyuki Nukina, Shoichi Ishiura, Vincent Lacroix, Amandine Campan-Fournier, Vincent Navratil, Emilie Chautard, Didier Auboeuf, Minoru Horie, Keiji Imoto, Kuang-Yung Lee, Maurice S. Swanson, Adolfo Lopez de Munain, Shin Inada, Hideki Itoh, Kazuo Nakazawa, Takashi Ashihara, Eric Wang, Thomas Zimmer, Denis Furling, Masanori P. Takahashi, Nicolas Charlet-Berguerand

**Affiliations:** 1 Department of Translational medicine and neurogenetics, IGBMC, CNRS UMR7104, INSERM U964, Université de Strasbourg, Illkirch 67400, France; 2 Sorbonne Universités UPMC Univ Paris 06, Inserm, CNRS, Centre de Recherche en Myologie UMRS974/FRE3617, Institut de Myologie, GH Pitié-Salpêtrière, Paris 75013, France; 3 Department of Neurology, Osaka University Graduate School of Medicine, Osaka 565-0871, Japan; 4 Department of Physiology, Friedrich Schiller University Hospital, Jena 07743, Germany; 5 Department of Bioinformatics and Molecular Neuropathology, Meiji Pharmaceutical University, Kiyose 205-8588, Japan; 6 Neuromuscular Research Center, Tampere University and University Hospital, Tampere 33520, Finland; 7 Department of Medical Genetics, Folkhälsan Institute of Genetics, Helsinki University, Helsinki 00250, Finland; 8 Department of Neurology, Vaasa Central Hospital, Vaasa 65130, Finland; 9 Department of Neurology, Stanford University, Stanford, California 94304, USA; 10 Service de Cardiologie, Université Paris-Descartes, Hôpital Cochin, AP-HP, Paris 75014, France; 11 Department of Neurology, Toneyama National Hospital, Toyonaka 560-8552, Japan; 12 CNRS UMR7175, Ecole Supérieure de Biotechnologies de Strasbourg, Illkirch 67400, France; 13 Division of Neurology, Hyogo Medical College, Nishinomiya 663-8501, Japan; 14 Laboratory of Structural Neuropathology, Doshisha University Graduate School of Brain Science, Kyoto 610-0394, Japan; 15 Graduate School of Arts and Sciences, University of Tokyo, Tokyo 153-8902, Japan; 16 Université Lyon 1, CNRS, UMR5558 LBBE, Villeurbanne 69622, France; 17 Hospices civils de Lyon, Laboratoire de cytogénétique constitutionelle, Bron 69500, France; 18 Pôle Rhône Alpes de Bioinformatique, Université Lyon 1, Bâtiment Gregor Mendel, Villeurbanne 69100, France; 19 Centre de Recherche en Cancérologie de Lyon, Lyon 69373, France; 20 Department of Cardiovascular and Respiratory Medicine, Shiga Medical University, Otsu 520-2192, Japan; 21 Department of Information Physiology, National Institute for Physiological Sciences, Okazaki 444-8585, Japan; 22 Department of Neurology, Chang Gung Memorial Hospital, Keelung 20401, Taiwan; 23 Department of Molecular Genetics and Microbiology, Center for NeuroGenetics and the Genetics Institute, University of Florida, College of Medicine, Gainesville, Florida 32610, USA; 24 Department of Neurology, Hospital Universitario DONOSTIA, Neuroscience Area, Institute Biodonostia CIBERNED and University of Basque Country UPV-EHU, San Sebastián 20014, Spain; 25 Laboratory of Biomedical Sciences and Information Management, National Cerebral and Cardiovascular Center Research Institute, Osaka 565-8565, Japan

## Abstract

Myotonic dystrophy (DM) is caused by the expression of mutant RNAs containing expanded CUG repeats that sequester muscleblind-like (MBNL) proteins, leading to alternative splicing changes. Cardiac alterations, characterized by conduction delays and arrhythmia, are the second most common cause of death in DM. Using RNA sequencing, here we identify novel splicing alterations in DM heart samples, including a switch from adult exon 6B towards fetal exon 6A in the cardiac sodium channel, *SCN5A*. We find that MBNL1 regulates alternative splicing of *SCN5A* mRNA and that the splicing variant of *SCN5A* produced in DM presents a reduced excitability compared with the control adult isoform. Importantly, reproducing splicing alteration of *Scn5a* in mice is sufficient to promote heart arrhythmia and cardiac-conduction delay, two predominant features of myotonic dystrophy. In conclusion, misregulation of the alternative splicing of *SCN5A* may contribute to a subset of the cardiac dysfunctions observed in myotonic dystrophy.

## Abstract (web summary)

Patients with myotonic dystrophy (MD) suffer from severe cardiac issues of unknown aetiology. Freyermuth *et al*. show that fatal changes in cardiac electrophysiological properties in humans and mice with MD may arise from misregulation of the alternative splicing of the cardiac Na^+^ channel *SCN5A* transcript, resulting in expression of its fetal form.

## Graphical abstract

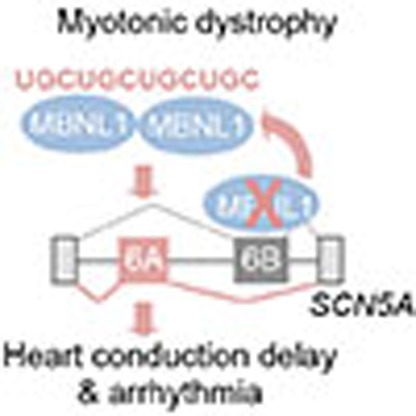

Myotonic dystrophy (DM), the most common adult-onset muscular dystrophy, includes two genetically distinct forms. DM of type 1 (DM1) and its severe congenital form (CDM1) are caused by an expansion of CTG repeats in the 3′-untranslated region (UTR) of the *DMPK* gene^1^,^2^,^3^. In contrast, DM of type 2 (DM2) is caused by an expansion of CCTG repeats within the first intron of the *CNBP* (also known as *ZNF9*) gene^4^.

The pathogenesis of DM involves a RNA gain-of-function mechanism caused by expression of mutant RNAs containing hundred to thousands of CUG or CCUG repeats that interfere with the splicing of other pre-mRNAs through dysfunction of two classes of RNA-binding proteins. MBNL proteins (MBNL1, MBNL2 and MBNL3) are sequestered within nuclear RNA foci formed by expanded CUG and CCUG repeats^5^,^6^, whereas expression and phosphorylation of CUG-binding protein 1 (CUGBP1, encoded by the *CELF1* gene) are increased in DM1 heart samples^7^. MBNL and CUGBP1 proteins regulate alternative splicing, and alterations of their functional levels in myotonic dystrophic tissues results in reversion to fetal splicing patterns for several mRNAs, such as the insulin receptor (*INSR*) (ref. 8), the muscle chloride channel (*CLCN1*) (refs 9, 10), dystrophin (*DMD*) (refs 11, 12) and key components of the skeletal muscle excitation–contraction coupling process, including amphiphysin2 (*BIN1*) (ref. 13), ryanodine receptor 1 (*RYR1*) (ref. 14), sarcoplasmic/endoplasmic reticulum Ca^2+^-ATPase SERCA1 (*ATP2A1*) (ref. 14) and the muscle calcium channel Ca_V_1.1 (*CACNA1S*) (ref. 15). Misregulation of the alternative splicing of the insulin receptor *INSR*, *CLCN1* and *DMD* mRNAs are associated with the insulin resistance^8^, myotonia^9^,^10^,^16^ and dystrophic process^12^, respectively, while alterations of the alternative splicing of *BIN1*, *RYR1*, *ATP2A1* and *CACNA1S* may contribute to the skeletal muscle weakness observed in DM^13^,^14^,^15^.

In contrast, the molecular mechanisms underlying the cardiac defects, which affect 80% of individuals with DM and represent the second most common cause of death in this disease^17^,^18^, are yet to be defined. Cardiac involvements in DM are characterized by cardiac-conduction delay that may result in fatal atrio-ventricular block, and by atrial or ventricular tachycardia^17^,^18^. Electrocardiography (ECG) analyses in DM patients indicate prolonged conduction time from the sinoatrial node to the ventricles (PR interval) and elongated ventricular depolarization (QRS duration). Interestingly, cardiac dysfunctions in DM are reminiscent in some aspect to an alteration of the cardiac sodium current. The α-subunit of the cardiac voltage-gated Na^+^ channel, Na_v_1.5, is encoded by the *SCN5A* gene and plays a key role in the excitability of cardiomyocytes and for rapid propagation of the impulse through the cardiac-conduction system. Mutations in *SCN5A* lead to a variety of arrhythmic disorders, including long QT3, progressive and non-progressive cardiac-conduction disease (also known as Lev-Lenègre disease), atrial fibrillation, sick sinus syndrome, Brugada syndrome and numerous overlapping syndromes^19^,^20^,^21^.

Using transcriptomic approaches, we identified various novel splicing changes in heart samples of DM1 individuals. Analysis of the RNA motifs enriched in the vicinity of these misregulated exons indicates that sequestration of the MBNL proteins is probably the main cause of splicing misregulation in heart of individuals with DM. Among these novel splicing alterations, we focused on misregulation of alternative splicing of the *SCN5A* pre-mRNA. This splicing alteration results in expression of a fetal isoform of *SCN5A* with altered electrophysiological properties. Of importance, we demonstrate that reproducing the splicing alteration of *Scn5a* in mouse is sufficient to cause heart arrhythmia and cardiac-conduction delay with elevated PR interval, which are key characteristics of the heart alterations observed in DM. These results suggest that altered splicing of *SCN5A* mRNA may participate to the electrical cardiac abnormalities observed in DM.

## Results

### Identification of splicing changes in DM heart samples

**Figure 1:**
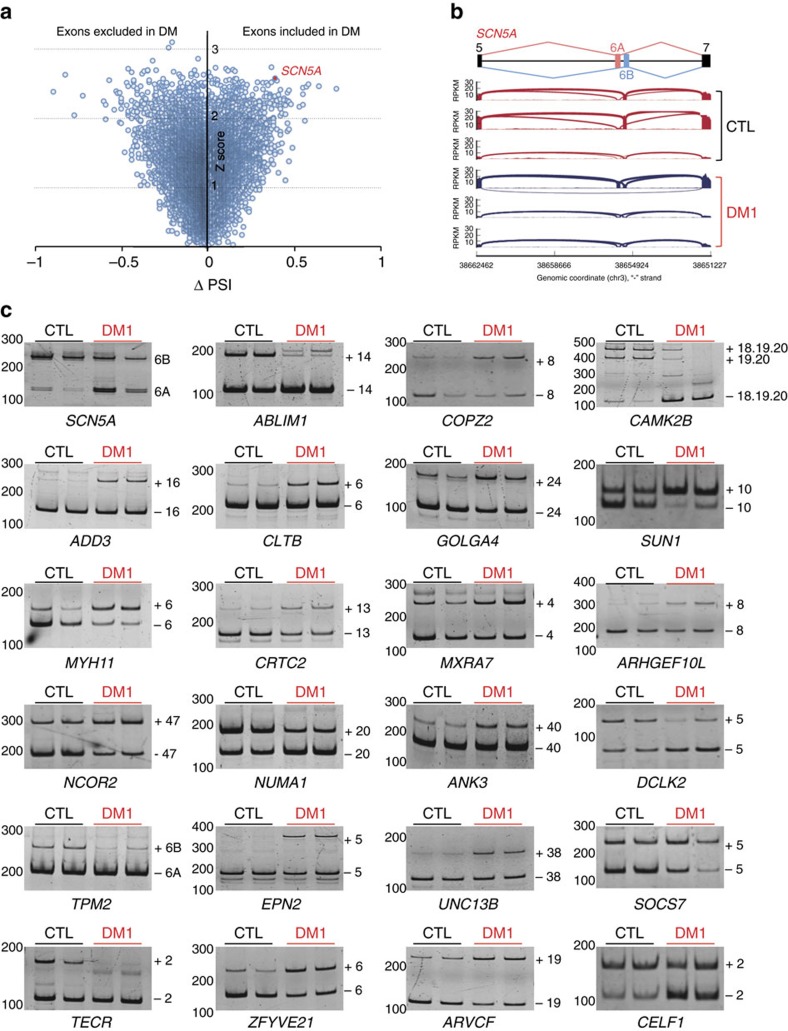
Identification of novel splicing misregulations in DM1 heart samples. (**a**) Δ-PSI versus Z-score plot of exon cassettes misregulations predicted by MISO analysis. (**b**) Exons structure and coverage of RNA-seq reads across *SCN5A* exons 5–7 show increased inclusion of exon 6A and decreased inclusion of exon 6B in heart samples of three DM1 patients (bottom, blue) versus three control samples (top, red). (**c**) Validation by RT–PCR of RNA-seq predictions in human heart samples of normal adult individuals (CTL, black) versus adult DM1 patients (DM1, red). Molecular size markers in bps are reported to the left of each RT–PCR gels. bp, base pair.

To determine novel splicing abnormalities in DM heart samples, we first used whole-genome microarrays (GeneChip Human Exon 1.0 ST array) on polyadenylated RNA extracted from left ventricle samples of three adult DM1 patients compared with three age-matched control individuals. Bioinformatic analyses predicted significant (Fold Change ⩾2, Sudent *t*-test, *P* value ≤0.01) changes in the splicing of 24 exons between control and DM1 samples (Supplementary Table 1), including a misregulation of the alternative splicing of the *SCN5A* pre-mRNA. To extend this analysis, we performed paired-end RNA sequencing (RNA-seq) on the same DM1 and control heart samples, yielding 1,611 million of mapped 100 bp reads. DESeq and Cuffdiff were then applied to estimate differential gene expression and over or under-expressed mRNAs were selected by using the Benjamini and Hochberg adjusted *P* values (false discovery rate (FDR) ≤0.1). A total of 9 and 19 upregulated genes were predicted differentially expressed with DESeq and Cuffdiff, respectively, but none were confirmed by quantitative real-time RT-qPCR analyses. This low number of differentially expressed mRNAs suggests that cardiac pathology in DM is not associated with drastic modifications of gene expression levels. In contrast, DEXSeq (ref. 22), which tests differential exon usage between two conditions, predicted 134 significant (Log2 Fold Change ⩾1.2, FDR ≤0.1) alternative splicing changes between control and DM1 heart samples (Supplementary Data 1). Similarly, MISO (ref. 23) analysis, which computes the fraction of mRNA that includes a given cassette alternative exon, predicted 259 significant (ΔPSI ⩾0.3; *Z*-score ⩾1.2) alternative splicing changes between control and DM1 heart samples (Fig. 1a and Supplementary Data 2), including a robust misregulation of the alternative splicing of *SCN5A* (Fig. 1b). MISO and DEXSeq predictions overlapped, but with some exceptions, such as the skipping of the consecutive exons 18, 19 and 20 of *CAMK2B* predicted by DEXSeq but not by MISO; or the shift of *SCN5A* exon 6B towards exon 6A identified by MISO but not by DEXSeq. These differences are inherent to their computation models, since MISO does not detect alterations of successive exons and DEXSeq does not identify mutually exclusive exons, highlighting that MISO and DEXSeq are complementary bioinformatics approaches. Next, we tested by PCR with reverse transcription (RT–PCR) forty candidate mRNAs having the highest probability of misregulation in DEXSeq and/or MISO analyses. We validated splicing alterations for 32 of them, including some that have been identified in previous studies (*TNNT2, TNNT3*, *ABLIM1, LDB3*, *MBNL1*, *CAMK2B*, *MAPT* and so on)^24^,^25^,^26^, and 20 others that represent, to the best of our knowledge, novel alterations of alternative splicing (*ADD3*, *GOLGA4*, *CRTC2*, *ARHGEF10L*, *ANK3*, *DCLK2*, *EPN2*, *UNC13B*, *TECR*, *ARVCF*, *SOCS7*, *CELF1* and so on) in DM1 heart samples (Fig. 1c). Of interest, some of these splicing alterations may be of pathological consequence in DM. For example, knockout of the *Socs7* gene in mouse results in insulin resistance^27^. Whether the splicing misregulation of *SOCS7* in DM contributes to insulin resistance remains to be tested. Also, RNA sequencing predicts an increased retention of the penultimate intron of *FCGRT*, which encodes the Fc fragment of the IgG receptor transporter α (FCRN) protein, involved in IGG recycling^28^. Whether splicing alteration of *FCGRT* in DM is responsible to the decreased level of IGG in blood of these patients is an attractive hypothesis that remains to be tested. Finally, RNA sequencing predicts misregulation of the alternative splicing of a cardiac-specific exon located in the 5′-UTR of *CELF1*, which encodes CUGBP1. Whether this alternative splicing may contribute to the increase levels of CUGBP1 protein observed in DM1 hearts remains also to be evaluated.

### Splicing altered in DM are enriched for MBNL-binding sites

**Figure 2:**
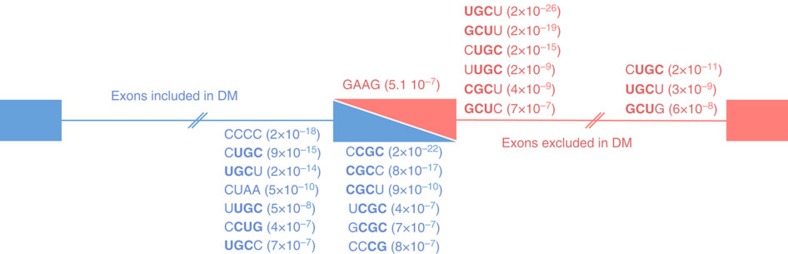
MBNL-binding motifs are enriched in vicinity of exons misregulated in DM1. Sequence and binomial test *P* values of 4-mer RNA motifs enriched downstream, within and upstream of exons misregulated in DM1 heart samples. Sequences enriched in exons excluded in DM are indicated in red, while sequences enriched in exons included in DM are indicated in blue.

Mutants RNAs containing expanded CUG or CCUG repeats interfere with the functional levels of CUGBP1 and MBNL proteins. Earlier studies determined that MBNL proteins bind to YGC RNA motifs (where Y is a pyrimidine)^29^,^30^,^31^,^32^, while CUGBP1 binds to UGU-enriched sequences^33^,^34^. To determine whether these RNA motifs are indeed present in the vicinity of exons misregulated in DM, we determined all 4-mer RNA motifs enriched within, upstream or downstream of the exons predicted as misregulated by MISO in DM1 heart samples, compared with 2,000 control exons (Fig. 2). Most RNA motifs significantly enriched (binomial test, *P* value <1.0 10^−7^) contained YGC sequences, while none were found to contain UGU sequences. Furthermore, YGC sequences were enriched upstream of exons abnormally included in DM1, while YGC motifs were enriched downstream of exons repressed in DM1. These results matched the MBNL splicing regulatory map determined by CLIP experiments, where binding of MBNL upstream of an exon tends to inhibit exon inclusion whereas binding of MBNL downstream of an exon generally stimulates exon inclusion^35^,^36^. In contrast, we found no enriched motifs for other RNA-binding proteins, including CUGBP1, rbFOX1, hnRNP H or Staufen. These results, as well as previous data^36^,^37^,^38^, support a model in which titration of MBNL proteins is the main cause of splicing change in DM1 heart, while misregulation of other RNA-binding proteins may contribute to a subset of splicing alterations.

### Splicing of *SCN5A* is misregulated in DM heart samples

**Figure 3:**
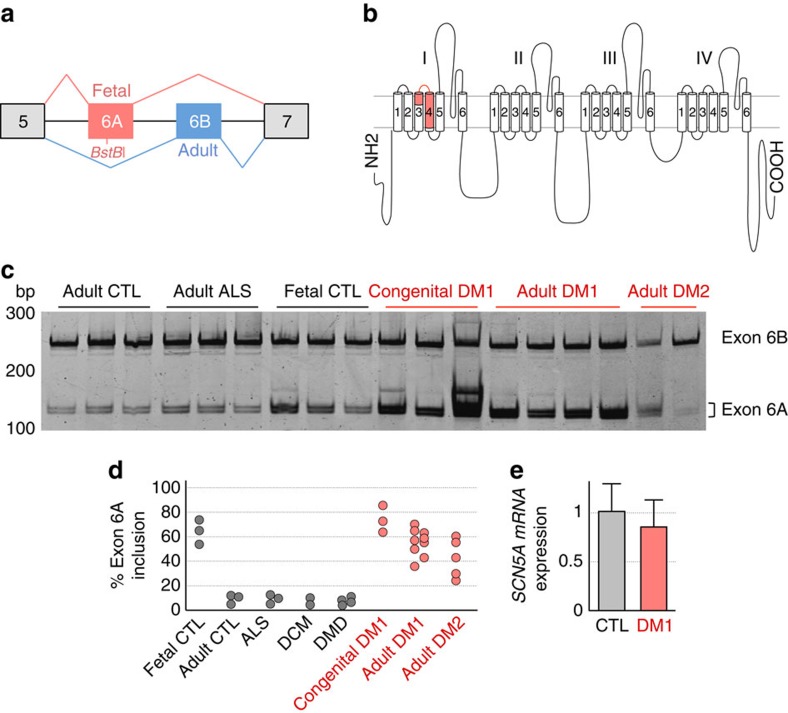
Splicing of *SCN5A* exon 6A is altered in DM heart samples. (**a**) Schematic representation of mutually exclusive exons 6A and 6B of *SCN5A*. *SCN5A* mRNA includes exon 6A (red) in fetal heart, while *SCN5A* mRNA expresses exon 6B (blue) in adult heart. (**b**) Schematic representation of SCN5A topology expressing exon 6A (red). Exons 6A or 6B encodes part of segment 3, connecting loop between S3 and S4 and most part of the voltage-sensitive segment 4 of domain 1 of the sodium channel SCN5A. (**c**). Representative *BstB*I-digested RT–PCR analysis of endogenous *SCN5A* mRNA from human heart samples of normal adult (CTL), adult ALS, non-DM fetuses (20, 24 and 35 weeks), congenital DM1 fetuses (CDM1 of 22, 25 and 28 weeks), adults DM1 and DM2 individuals. Molecular size marker is indicated in bp. (**d**) Graphical representation of RT–PCR analysis depicting the percentage of *SCN5A* mRNA including exon 6A in left ventricular heart samples from fetal and adult control, ALS, DCM, DMD, CDM1 and adult DM1 and DM2 individuals. (**e**) Graphical representation of quantitative real-time RT-qPCR depicting the mRNA expression of *SCN5A* relative to *RPLP0* in control normal adults (*n*=5) versus adult DM1 (*n*=5) heart samples. Bars indicate s.e.m. bp, base pairs.

Both microarray and RNA-seq predicted misregulation of alternative splicing of *SCN5A* pre-mRNA in DM1 heart samples. Splicing of *SCN5A* is developmentally regulated, such that exon 6A is included in fetal heart but rapidly replaced by exon 6B after birth^39^. Consequently, *SCN5A* exon 6A is named as embryonic or fetal, while exon 6B is known as adult. Exons 6A and 6B are mutually exclusive exons encoding part of the voltage sensor, segments 3 and 4 located in the domain I of the sodium channel (Fig. 3a,b). These are key segments for the electrical activity of the sodium channel, and inclusion of either fetal exon 6A or adult exon 6B results in channel isoforms, named, respectively, hNa_v_1.5e and hNa_v_1.5, with different electrophysiological properties^39^,^40^,^41^. We confirmed our microarray and RNA-seq predictions by RT–PCR and found that adult *SCN5A* exon 6B is partly replaced by its fetal exon 6A in heart samples of individuals with DM, including adult DM1 and adult DM2 cases (Fig. 3c). Note that to differentiate exon 6B from exon 6A that have the exact same length of 92 bp, we took advantage of a *Bstb*I restriction site present only in exon 6A, which thus appears as a *Bstb*I-digested doublet band in Fig. 3c. These results are consistent with the recent report of a splicing misregulation of *SCN5A* in one DM1 heart sample^42^. Although splicing of *SCN5A* is misregulated in DM1, we observed no correlation between the percentage of *SCN5A* exon 6A inclusion and the increased duration of the PR interval and only a very limited, if any, correlation between misregulation of *SCN5A* exon 6A splicing and alteration of the QRS duration in individuals with DM1 (*R*^2^ of 0.2 with six DM1 samples; Supplementary Fig. 1). Misregulation of *SCN5A* splicing was specific to DM, as we did not observe inclusion of exon 6A in heart samples from individual affected with Duchenne muscular dystrophy (DMD), amyotrophic lateral sclerosis (ALS) or dilated cardiomyopathy (DCM) (Fig. 3d). Moreover, misregulation of splicing in DM1 was specific and not global, as we observed no splicing changes of *SCN5A* alternative exon 18, of *CACNA1C* mutually exclusive exons 8A and 8B, of *KCNAB1* alternative exons 2 and 11, or of *KCNQ1* alternative exons 2 and 5 (Supplementary Fig. 2). Finally, we observed no significant alteration of the expression level of *SCN5A* mRNA by quantitative real-time RT-qPCR (Fig. 3e). Overall, these results indicate a specific misregulation of alternative splicing of *SCN5A* resulting in expression of a fetal form of this channel in adult DM heart. These results are consistent with previous studies where alternative splicing changes in DM resume a MBNL-dependent fetal splicing pattern that persist in adult tissues^26^,^37^.

### Alternative splicing of *SCN5A* is regulated by MBNL1

**Figure 4:**
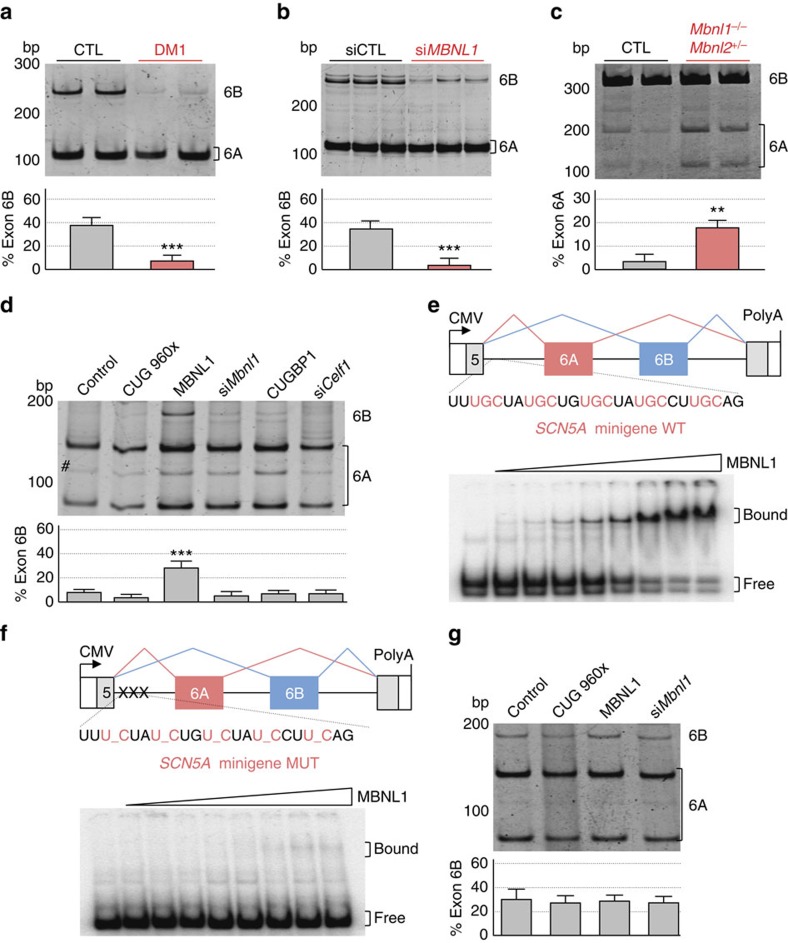
MBNL1 regulates alternative splicing of *SCN5A*. (**a**) Upper panel, RT–PCR analysis of endogenous *SCN5A* mRNA from differentiated primary muscle cell cultures derived from biopsies of control or DM1 individuals. (lower) Quantification of the percentage of *SCN5A* mRNA including exon 6B. (**b**, upper) RT–PCR analysis of endogenous *SCN5A* mRNA from human differentiated cultures of control primary muscle cells transfected with a scrambled siRNA (siCTL) or a siRNA targeting *MBNL1* mRNA (si*MBNL1*). (lower) Percentage of *SCN5A* mRNA including exon 6B. (**c**, upper) RT–PCR analysis of endogenous *Scn5a* mRNA in heart samples of wild-type and compound *Mbnl1*^−/−^, *Mbnl2*^+/−^ double knockout mice. (lower) Percentage of *Scn5a* mRNA including exon 6A. (**d**, upper) RT–PCR analysis of exogenous *SCN5A* mRNA from differentiated C2C12 muscle cells co-transfected with a *SCN5A* minigene containing exons 6A and 6B bordered by their introns and with either a plasmid expressing 960 CTG repeats, MBNL1, CUGBP1 or with a siRNA directed against *Mbnl1* (si*Mbnl1*) or *Celf1* (encoding Cugbp1; si*Celf1*). # Indicates usage of a cryptic splice site inherent to the minigene. (lower) Percentage of *SCN5A* mRNA including exon 6B. (**e**, upper) Schematic representation of *SCN5A* minigene, including the UGC-rich sequence used for binding assays. (lower) Gel-shift assays were performed using 5–1,000 nM of purified bacterial recombinant GST-MBNL1Δ^101^ and a uniformly ^32^P-CTP labelled RNA. (**f**, upper) Schematic representation of mutant *SCN5A* minigene, including the mutant sequence, used for binding assays. (lower) Gel-shift assay performed as in **e**. (**g**, upper) RT–PCR analysis of exogenous *SCN5A* mRNA from differentiated C2C12 muscle cells co-transfected with mutant *SCN5A* minigene and with a plasmid expressing 960 CTG repeats or MBNL1 or with a siRNA directed against *Mbnl1* (si*Mbnl1*). (lower) Percentage of *SCN5A* mRNA including exon 6B. All transfection and gel-shift experiments were repeated three to five times. Molecular size markers are indicated in bp. Bars indicate s.e.m. Student test, ** indicates *P*<0.01, *** indicates *P*<0.001. bp, base pairs.

To determine the mechanisms underlying misregulation of *SCN5A* splicing, we first determined its splicing pattern in cell models of DM. Since *SCN5A* is expressed at low level in culture of immature skeletal muscle cells, we investigated its splicing in primary cultures of differentiated skeletal muscle cells originating from muscle biopsies of control and DM1 individuals. RT–PCR experiments determined a switch of exon 6B towards exon 6A in DM1 muscle cells compared with control, reproducing the splicing alteration observed in cardiac tissue (Fig. 4a). Of technical interest, the basal level of exon 6A inclusion was higher in muscle cell cultures than in adult heart samples (compare Fig. 4a to Fig. 3c), which probably reflect the immature aspect of cell cultures. Since mutant RNAs containing expanded CUG or CCUG repeats interfere with alternative splicing through titration of MBNL proteins, we tested whether MBNL1 regulates *SCN5A* splicing. Reduction of *MBNL1* expression through a siRNA-mediated approach in human control primary muscle cells mimicked the effect of CUG repeats and promoted a switch from adult exon 6B towards fetal exon 6A (Fig. 4b). Western blotting analysis confirmed the successful depletion of MBNL1 expression (Supplementary Fig. 3A). Next, we assessed alternative splicing of *Scn5A* in heart samples of *Mbnl* knockout mice^43^. RT–PCR analysis shows that inclusion of the exon 6A of *Scn5a* is increased in heart samples of mice with no Mbnl1 and reduced level of Mbnl2 (*Mbnl1*^−/−^, *Mbnl2*^+/−^)(Fig. 4c). The increased inclusion of *Scn5a* exon 6A in *Mbnl* knockout mice is significant (Student *t*-test, *P* value ≤0.01) but rather mild, probably reflecting difference in regulation of alternative splicing between human and mouse or the compensatory effect of residual Mbnl2 expression^43^. This hypothesis is consistent with the mild splicing alteration of *Scn5A* observed in the sole *Mbnl1* knockout mice^44^. Overall, these results suggest that MBNL proteins regulate the alternative splicing of *SCN5A* exons 6A and 6B. To determine whether this regulation is direct or indirect, we constructed a minigene containing exons 6A and 6B of *SCN5A* bordered by their intronic regions. Expression of this construct in mouse C2C12 myoblasts reproduced a fetal pattern with mainly inclusion of exon 6A (Fig. 4d). Since inclusion of exon 6B was repressed, reduction of Mbnl1 activity through siRNA or expression of expanded CUG repeats had no further repressive effect on exon 6B. In contrast, expression of MBNL1 promoted a switch from fetal exon 6A towards adult exon 6B, while expression or siRNA-mediated depletion of CUGBP1 had no effect (Fig. 4d). Western blotting analysis confirmed that siRNA transfection efficiently reduced endogenous Mbnl1 or Cugbp1 expression (Supplementary Fig. 3B and C). Next, gel-shift assays determined that recombinant purified GST-tagged MBNL1 bound to UGC RNA motifs located upstream of exon 6A (Fig. 4e). Of interest, this UGC sequence is absent from the mouse genome, which may explain the mild splicing alteration of *Scn5A* observed in mice knockout for Mbnl proteins. Mutation of these UGC motifs abolished MBNL1 binding (Fig. 4f), as well as the regulatory effect of MBNL1 on a mutant *SCN5A* minigene (Fig. 4g). Overall, these results establish that MBNL1 regulates directly alternative splicing of *SCN5A* exons 6A/6B.

### *SCN5A* splicing forms present different electrical properties

**Figure 5:**
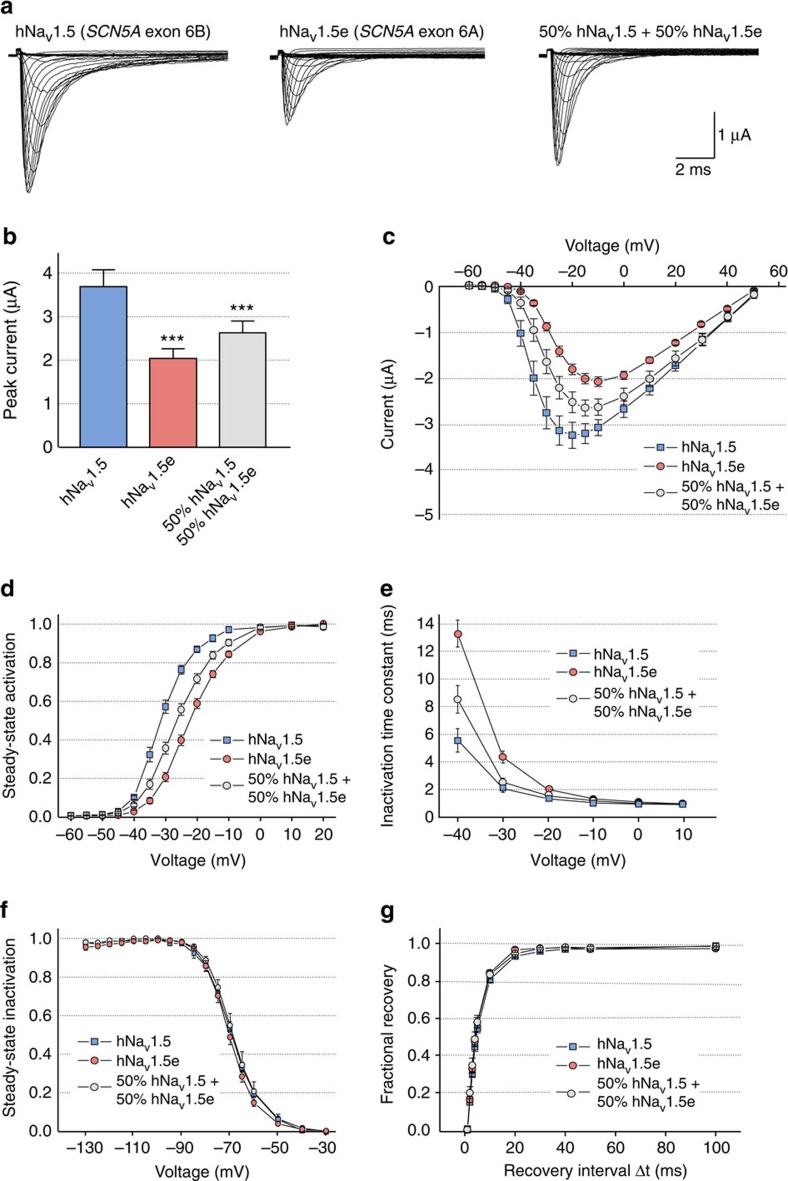
Electrophysiological properties of hNa_v_1.5 and hNa_v_1.5e channels. (**a**) Representative Na^+^ currents generated in *Xenopus* oocytes by hNa_v_1.5 (encoded by *SCN5A* containing the adult exon 6B), hNa_v_1.5e (encoded by *SCN5A* including the fetal exon 6A), and simultaneously expressed Na_v_1.5 and Na_v_1.5e channels at a 1:1 ratio. (**b**) Peak current amplitudes at the test potential of −10 mV in *Xenopus* oocytes injected with equimolar amount of cRNA encoding hNa_v_1.5, hNa_v_1.5e or 1:1 combination of Na_v_1.5 and Na_v_1.5e channels. (**c**) Current–voltage relationships. (**d**) Steady-state activation curves. (**e**) Inactivation time constants *τ*h (ms) at different test pulses. (**f**) Steady-state inactivation curves. (**g**) Fractional recovery curves. Data were obtained from 11 different batches of oocytes. To illustrate steady-state activation, steady-state inactivation and recovery from inactivation, we used 3–5 representative measurements. For total number of measurements (*n*=25–27) and for statistical data evaluation (Vm, s) see the Supplementary Table 2. Bars indicate s.e.m. Student test, *** indicates *P*<0.001.

*SCN5A* encodes Na_v_1.5, the main cardiac voltage-gated sodium channel, and loss-of-function mutations in *SCN5A* lead to a variety of arrhythmic disorders, which share some common pathological features with DM. Furthermore, exons 6A and 6B differ at seven amino acid positions, resulting in channel variants with different electrophysiological properties^39^,^40^,^41^. To investigate the consequences of the switch from *SCN5A* exon 6B towards exon 6A observed in DM, we first examined in *Xenopus* oocytes the sodium currents generated by either hNa_v_1.5e, the splice variant of *SCN5A* containing the fetal exon 6A, or hNa_v_1.5, encoded by *SCN5*A containing the adult control exon 6B (Fig. 5a and Supplementary Table 2). Injection of RNA encoding hNav1.5e, which is the splicing isoform of SCN5A found in DM, indicated a significant reduction of the sodium current amplitude of 45%, compared with hNa_v_1.5, the normal adult *SCN5A* exon 6B form (Fig. 5b,c). Since, the extent of splicing misregulation varies among DM individuals, which typically express a mix of SCN5A splicing forms containing either exon 6A or exon 6B (cf. Fig. 3c), we analysed sodium currents generated by a mix of both *SCN5*A isoforms (Fig. 5a). Injecting *Xenopus* oocytes with an equimolar mix of RNA encoding each channel, namely 50% of hNa_v_1.5e (*SCN5A* containing fetal exon 6A) and 50% of hNa_v_1.5 (SCN5A expressing adult exon 6B), resulted in a reduction of 30% of the current amplitude compared with the control hNa_v_1.5 (Fig. 5b,c). Next, two-electrode voltage clamp recording experiments revealed that the steady-state activation of the fetal hNa_v_1.5e was shifted by 7 mV towards depolarized potential compared with the control adult hNa_v_1.5 form (Fig. 5d and Supplementary Table 2). This shift is consistent with the shift observed previously in transfected mammalian cells^39^,^40^,^41^, thus validating our approach in *Xenopus* oocytes. To better reproduce the situation observed in DM, we injected in *Xenopus* oocytes an equimolar mix of DM (hNa_v_1.5e, fetal exon 6A) and control (hNa_v_1.5, adult exon 6B) RNA isoforms of *SCN5A*. Importantly, this mix of splicing forms also presented a significant shift of steady-state activation towards depolarized potentials by 3.8 mV, compared with the control hNa_v_1.5 form (Fig. 5d, Supplementary Table 2). Correspondingly, a similar shift was observed for the time constant of inactivation (Fig. 5e). Consistent with previous electrophysiological studies^39^,^40^,^41^, no significant differences were observed between hNa_v_1.5 and hNa_v_1.5e regarding steady-state inactivation and recovery from inactivation (Fig. 5f,g). Overall, our results are consistent with previous studies^39^,^40^,^41^, and demonstrate that hNa_v_1.5e, the splicing form of *SCN5A* expressed in DM and containing the fetal exon 6A, presents a reduced excitability compared with hNa_v_1.5, which is the adult control *SCN5A* isoform containing exon 6B.

### Alteration of *SCN5A* splicing leads to heart conduction defects

**Figure 6:**
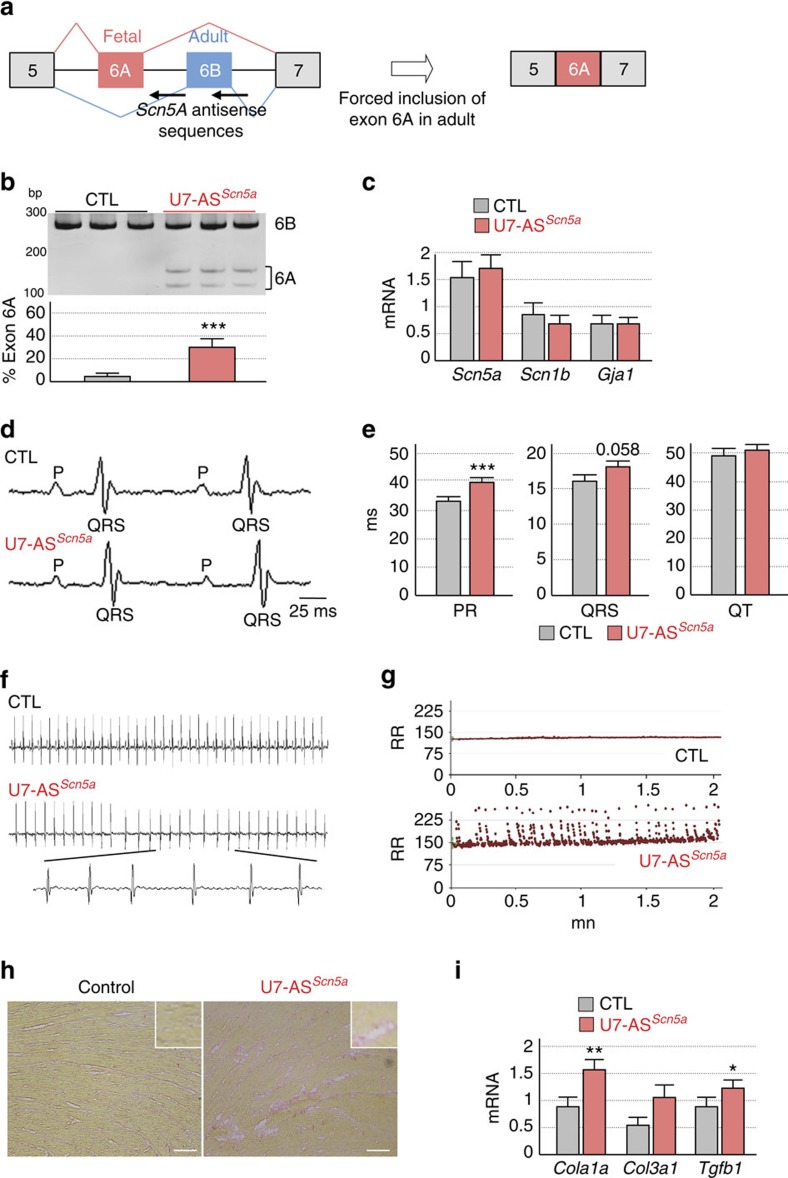
Alteration of *Scn5a* splicing causes heart conduction defects and arrhythmias. (**a**) Schematic representation of mutually exclusive exons 6A and 6B of *Scn5a* and of antisense sequences driven by optimized U7-snRNAs (U7-AS^*Scn5a*^) to force fetal exon 6A inclusion in adult wild-type mouse heart. (**b**, upper) RT–PCR analysis of the alternative splicing of endogenous *Scn5a* mRNA from heart samples of mice injected with AAV2/9 expressing U7-AS^*Scn5a*^ compared with control injected mice. Molecular size marker is indicated in bp. (lower) Percentage of *Scn5a* mRNA including exon 6A. (**c**) Real-time RT-qPCR quantification of the expression of *Scn5a, Scn1b* and *GJja1* (connexin 43) mRNAs in heart samples of mice expressing U7-AS^*Scn5a*^ (*n*=6) compared with control injected mice (*n*=6). (**d**) Representative ECG traces show prolongation of the PR interval in U7-AS^*Scn5a*^-injected mice compared with control mice. (**e**) ECG measures of PR interval, QRS and QT intervals in 4-month-old mice injected with AAV2/9 expressing U7-AS^*Scn5a*^ (*n*=25) compared with age-matched control mice (*n*=17). (**f**) Representative ECG traces reveal atrial fibrillation in U7-AS^*Scn5a*^-injected mice compared with control mice. (**g**) Variation of the RR interval indicates evidences of heart arrhythmias in U7-AS^*Scn5a*^-injected mice (*n*=25) compared with control mice (*n*=17). (**h**) Representative image of six analysed heart samples showing mild fibrosis revealed by Red Sirius histology staining in AAV-U7-AS^*Scn5a*^-injected mice. Scale bar, 100 μm. (**i**) Real-time RT-qPCR quantification of the expression of *Cola1a, Col3a1* and *Tgfb* mRNAs in heart of control (*n*=6) or AAV-U7-AS^*Scn5a*^-injected mice (*n*=6). Bars indicate s.e.m. Student test, * indicates *P*<0.5, ** indicates *P*<0.01, *** indicates *P*<0.001. bp, base pair.

**Figure 7:**
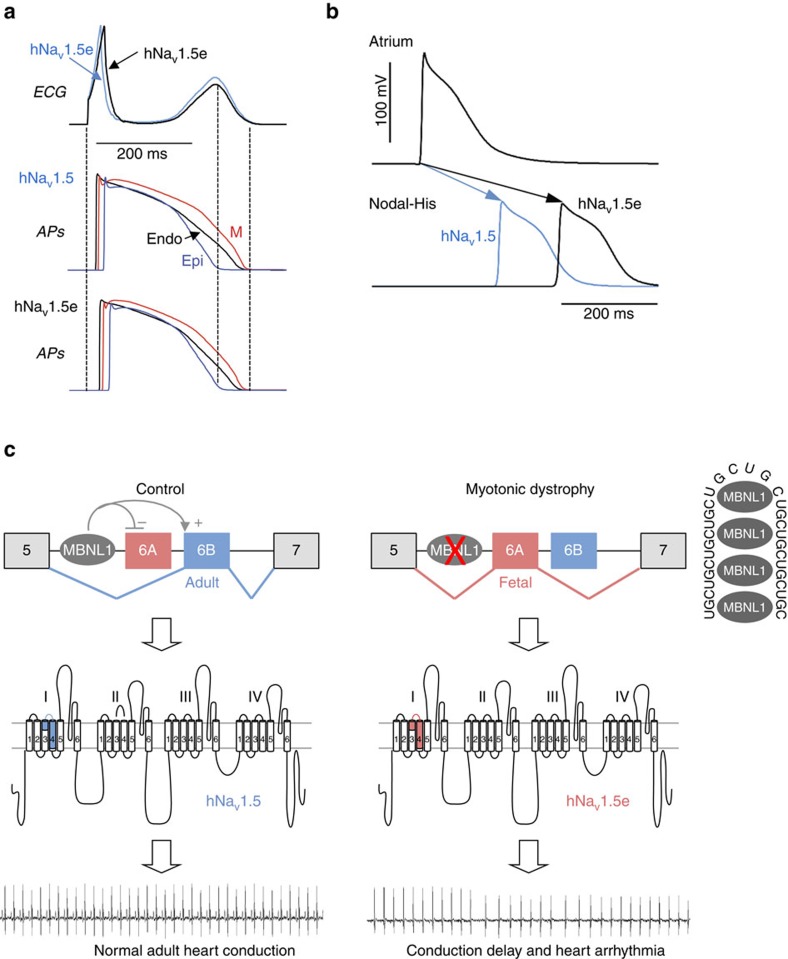
Computer simulation predicts cardiac conduction abnormalities in DM. (**a**) Simulations of ECG (upper) and action potential (AP) alterations (middle and lower) caused by the switch from adult *SCN5A* exon 6B towards fetal exon 6A, using a modified human ventricular ORd model. (**b**) Simulations of the atrio-ventricular changes caused by inclusion of SCN5A fetal exon 6A instead of adult exon 6B, employing an atrio-ventricular nodal model. APs of atrial and AV node cells were simulated and atrium-His interval was measured as the difference in the latency of APs between atrial and nodal-His cells. (**c**) Model of splicing alteration of the cardiac sodium channel, *SCN5A*, in DM. MBNL proteins regulate the switch from *SCN5A* exon 6A in fetal heart to exon 6B in adult. In DM, titration of MBNL proteins by mutant RNA containing expanded CUG repeats leads to expression of a fetal splicing form of *SCN5A*, inappropriate to adult heart physiology, ultimately resulting in cardiac-conduction delay and heart arrhythmias, which are two keys features of DM.

Misregulation of the alternative splicing of *SCN5A* in DM is one alteration identified among many others, thus questioning the contribution of *SCN5A* misregulation to the cardiac symptoms observed in DM. To test the physiological importance of *SCN5A* splicing misregulation, we artificially forced the switch from adult exon 6B towards fetal exon 6A into wild-type adult mouse heart using an exon-skipping strategy (Fig. 6a). To insure efficient transduction of the cardiac muscle and continuous expression of nuclear antisense oligonucleotides, we engineered and produced adeno-associated virus (AAV2/9) expressing optimized U7-snRNA fused to *Scn5a* antisense sequences (U7-AS^*Scn5a*^). Splicing analysis revealed that combination of two U7-AS constructs, spanning intron 6/exon 6B junction and exon 6B of *Scn5A*, promoted a switch from inclusion of adult exon 6B towards inclusion of the fetal exon 6A (Supplementary Fig. 4A). Thus, AAV2/9 expressing both U7-AS constructs (AAV-U7-AS^*Scn5a*^) were injected systemically into newborn wild-type mice and cardiac functions were investigated 4 and 6 months post injection. Control animals injected either with saline or empty AAV2/9 presented no splicing alterations of *Scn5a* and normal cardiac functions. In contrast, mice injected with AAV-U7-AS^*Scn5a*^ presented a decreased inclusion of adult exon 6B with a concomitant 30–40% increase of the inclusion of exon 6A, thus reproducing the situation observed in DM (Fig. 6b). Quantitative RT–PCR demonstrated no changes in the expression of *Scn5a* mRNA or of its associated subunit *Scn1b* between control- and AAV-U7-AS^*Scn5a*^-injected mice (Fig. 6c). Importantly, AAV-U7-AS^*Scn5a*^-injected mice reproduce some of the key pathological features of DM, including conduction defects and heart arrhythmias. Indeed, ECG performed 4 months post injection revealed a significant prolongation of the PR intervals (Student *t*-test, *P* value ≤0.001) in AAV-U7-AS^*Scn5a*^-injected mice compared with control injected mice (Fig. 6d,e). In contrast, QT interval was not significantly altered, and we identified only a trend towards increased QRS duration (Student *t*-test, *P* value of 0.058 with 8 AAV-U7-AS^*Scn5a*^-injected mice on 25 presenting a QRS higher than 19 ms versus 16.5 ms in control mice) (Fig. 6e and Supplementary Fig. 4B). Similarly, analysis of heart functions in 6-month-old animals showed that AAV-U7-AS^*Scn5a*^-injected mice present a consistent increase of the PR interval compared with control injected mice (40.5 ms versus 34.8 ms respectively; Student *t*-test, *P* value ≤0.05), without significant changes of the QRS and QT intervals (Supplementary Fig. 4B). Of interest, a similar elongation of the PR interval was observed in *Scn5a*^+/−^ mice, which are hemizygote for *Scn5a* expression and represent an established model for cardiac-conduction disease^45^,^46^,^47^. Furthermore, ECG analyses also revealed that 44% of AAV-U7-AS^*Scn5a*^-injected mice develop significant (Student *t*-test, *P*<0,001) heart arrhythmia at 4 months post injection with an average of five arrhythmic events, defined as variation of the RR interval, per minute whereas control injected animals showed no alterations (Fig. 6f,g). We did not detect ventricular fibrillations or second and third-degree heart blocks in any injected animals. In contrast, we observed supraventricular premature contractions and atrial fibrillation in AAV-U7-AS^*Scn5a*^-injected mice (Fig. 6f), and five of these injected mice died suddenly between 4 and 6 months post injections (none of the control mice died). These electrical alterations were specific and not caused by global cardiac remodelling since we observed neither systolic nor diastolic alterations by doppler echocardiography (Supplementary Table 3) and no change in heart/body weight ratio (4.4±0,1 mg g^−1^ in control, *n*=9, versus 4,7±0,2 mg g^−1^ in AAV-U7-AS^*Scn5a*^-injected animals, *n*=14). As further control, H&E-staining revealed normal heart structures with no evident cardiomyopathy or dilation at 6 months post AAV injections (Supplementary Fig. 4C). Similarly, quantitative RT–PCR experiments show no alteration in the expression levels of *Nppa*, *Nppb* (encoding Anp and Bnp, respectively) and *Myh7* mRNAs (Supplementary Fig. 4D), suggesting no overt cardiac remodelling in antisense AAV-U7-AS^*Scn5a*^-injected mice. Moreover, Sirius Red staining confirmed normal heart structures but also revealed some mild fibrosis (Fig. 6h), which was confirmed by increased expression of collagen *Cola1a* and *Tgfb1* mRNAs (Fig. 6i). Interestingly, mild fibrosis is also observed in DM cardiac samples^17^,^18^, as well as in individuals and mice models with loss-of-function mutations of the *SCN5A* gene^20^,^21^,^46^,^47^. Overall, heart arrhythmias and prolonged PR interval in AAV-U7-AS^*Scn5a*^-injected animals demonstrate that inclusion of the fetal exon 6A of *Scn5a* is inappropriate to adult mouse heart physiology. However, while we found a clear elongation of the PR interval, we did not detect a significant alteration of the QRS duration as only a third of AAV-U7-AS^*Scn5a*^-injected mice present increased QRS duration (>19 ms). Interestingly, similar findings have been described in *Scn5a*^+/−^ mice, which all show elongation of the PR interval, while only a subset of *Scn5a*^+/−^ animals present a prolongation of the QRS interval^45^. Hence, elongation of the PR interval is not systematically associated with increased duration of the QRS in mouse model of Scn5a dysfunction. Thus, to strengthen our data, we mathematically tested whether human cardiac parameters would be altered by the electrophysiological differences caused by the switch from adult exon 6B towards fetal exon 6A of *SCN5A*. Simulation based on a modified O'Hara-Rudy model^48^,^49^ predicted a change of the QRS duration from 72 ms with control adult hNa_v_1.5 to 88 ms with fetal hNa_v_1.5e, hence a 22% increase (Fig. 7a and Supplementary Fig. 5). Furthermore, we also tested extent of atrio-ventricular change^50^. Mathematical simulation predicted a change of the atrium-His interval from 81 ms with control hNa_v_1.5 to 143 ms with fetal hNa_v_1.5e (Fig. 7b). Overall, these results support our mouse results and provide additional evidences that misregulation of *SCN5A* alternative splicing causes cardiac-conduction abnormalities, which is a key pathological feature of DM (Fig. 7c).

## Discussion

Cardiac defects affect 80% of individuals with DM and represent the second most common cause of death in this disease^17^,^18^. However, the molecular mechanisms responsible for cardiac-conduction delay and ventricular tachycardia in DM are unclear. Using RNA sequencing we identified various novel splicing misregulation events in DM1 heart samples. Among these changes, the splicing switch from adult exon 6B to fetal exon 6A in *SCN5A* mRNA is of particular interest. Previous studies^39^,^40^,^41^ as well as ours indicate that hNa_v_1.5e, the splicing variant of *SCN5A* found in DM and that contains the fetal exon 6A, possesses a reduced excitability compared with the normal adult splicing form of *SCN5A* containing the exon 6B. Consequently, the switch from the hNa_v_1.5 to the hNa_v_1.5e channel in DM may cause a slower upstroke velocity of the cardiac action potential, leading to conduction slowing. Importantly, this hypothesis is supported by mathematical simulation as well by animal model, since imposing a switch from inclusion of the control adult exon 6B towards using the fetal exon 6A of *Scn5A* in adult mouse heart led to cardiac-conduction delay and heart arrhythmias, two key features of DM. Moreover, clinical evidence also supports an alteration of the sodium current in DM. Indeed, the electrophysiological features^39^,^40^,^41^ of the fetal isoform of *SCN5A* expressed in DM are similar to the electrophysiological characteristics observed with loss-of-function mutations of *SCN5A* causing cardiac-conduction disease^51^,^52^,^53^. Also, there are some similarities of ECG recording, including prolongation of the PR interval and of the QRS duration, between individuals with DM and individuals affected by cardiac-conduction disease caused by loss-of-function mutations in *SCN5A*^42^,^54^. Finally, the induction of abnormal ECG pattern in DM patients treated with ajmaline^55^,^56^, a class Ia antiarrhythmic agent acting on the cardiac sodium channel and the abnormal sodium current observed in a mouse model of DM^57^, are also evocative of a dysfunction of the sodium channel in DM. Overall, our results suggest that misregulation of the splicing of *SCN5A* participates in a subset of electrical cardiac alterations observed in DM, namely the cardiac-conduction delay and the heart arrhythmias. However, it is likely that other alternative splicing alterations and/or mechanisms^58^,^59^,^60^,^61^ are participating to the full pattern of cardiac alterations in DM since knockout of *Mbnl1* and *Mbnl2* in mice leads to only mild alteration of *Scn5A* splicing, while these mice show severe conduction disease and cardiac dilatation^43^,^44^.

In conclusion, this work may also have some clinical importance such as considering with caution the treatments of DM patients with pharmaceutical agents that reduce the activity of the cardiac sodium channel, including mexiletine, flecainide and other antiarrhythmic drugs of class I. In that aspect, this study may provide a molecular explanation to the adverse cardiac reaction of some patients with myotonic dystrophic to treatment with drugs reducing activity of SCN5A (refs 62, 63). Involvement of the cardiac sodium channel in DM might also highlight the importance of considering polymorphism in the *SCN5A* gene, as well as in other genes such as *SCN10A*, as a possible cause of the clustering of cardiac alterations in some families of DM^64^, an hypothesis supported by the recent report that DM exacerbates Brugada syndrome caused by a mutation in *SCN5A* (ref. 65). Inversely, it remains to be tested whether mutations in intronic regions regulating alternative splicing of *SCN5A* exon 6A/6B might be considered as a cause of cardiac-conduction abnormalities in patients in whom no mutations were identified in the coding sequence of *SCN5A*. Also, if dysfunction of the cardiac sodium channel in DM is comparable in some aspects to cardiac-conduction disease caused by mutations in *SCN5A*, an attractive supposition would be that therapeutic approaches developed for these diseases could be considered for DM. Finally, if cardiac-conduction defects in DM are associated with an alteration of the alternative splicing of *SCN5A*, one may hope that correction of splicing misregulations through approaches releasing MBNL1 from CUG expanded repeats or through approaches reducing the expression of pathogenic CUG RNA^66^,^67^ may alleviate the cardiac symptoms of DM.

## Methods

### Human samples

All samples were heart left ventricles that were sampled with the informed consent of individuals and approved by the Institutional Review Board of the Pitié-Salpêtrière hospital, of the Neuromuscular Research Center of the Tampere University Hospital, of the Hospital Donostia and of the Toneyama National Hospital. Non-affected heart samples (CTL #1 to #3) were purchased at Ambion and Stratagene, respectively. DCM #1 and #2 were patients suffering from DCM of uncharacterized genetic origin. DMD #1 to #4 were patients affected with DMD. ALS #1 to #3 were patients with ALS described previously^68^. DM1 samples #1 and 2 were from cardiac transplantation for end stage heart failure with conduction system disease in a 45-year-old male and a 48-year-old female suffering from DM1. DM1 patient #3 is a 58-year-old female with 500 CTG repetitions in blood. DM1 patients #4 to #6 have been described previously^68^ with expansion of 4,300 (female, 58 years); 4,800 (male, 63 years); 5,800 (female, 56 years); and ∼6,000 CTG repeats in heart. DM1 samples #7 to #9 were patients with BAV1 and sadly unknown number of CTG repeats. DM2 patients #1 to 4 were described previously^69^,^70^. DM2 patients #5 heart sample was obtained at immediate autopsy after sudden bilateral renal thrombosis at the age of 71 years. He had ∼4,800 CCTG repeats in leucocytes.

### mRNA sequencing

RNA sequencing was performed on DM1 samples #4 to #6 described previously^68^. After isolation of total cellular RNA from human heart samples, libraries of template molecules suitable for high throughput DNA sequencing were created using ‘TruSeq RNA Sample Preparation v2 Kit' (Illumina). Briefly, mRNA was purified from 1 μg total RNA using poly-T oligo-attached magnetic beads and fragmented using divalent cations at 94 °C for 8 min. The cleaved mRNA fragments were reverse transcribed to cDNA using random primers then the second strand of the cDNA was synthesized using DNA Polymerase I and RNase H. The double-stranded cDNA fragments were blunted using T4 DNA polymerase, Klenow DNA polymerase and T4 PNK. A single ‘A' nucleotide was added to the 3′ ends of the blunt DNA fragments using a Klenow fragment (3′- to 5′-exo minus) enzyme. The cDNA fragments were ligated to double-stranded adapters using T4 DNA Ligase. The ligated products were enriched by PCR amplification (30 s at 98 °C; (10 s at 98 °C, 30 s at 60 °C, 30 s at 72 °C) × 12 cycles; 5 min at 72 °C). Then surplus PCR primers were removed by purification using AMPure XP beads (Agencourt Biosciences Corporation). DNA libraries were checked for quality and quantified using 2100 Bioanalyzer (Agilent). The libraries were loaded in the flow cell at 7pM concentration and clusters were generated in the Cbot and sequenced in the Illumina Hiseq 2000 as paired-end 2 × 100 base reads. FASTQ files were generated with CASAVA v1.8.2.

### Bioinformatic analysis

A total of 1.857.442.522 short reads were mapped onto the human genome (hg19 assembly) by using Tophat2 software^71^ (release v2.0.5) with a mapping rate ranging from 83 to 90%. Htseq-count (release 0.5.3p3). The Cufflinks software^72^ (release 2.0.2) was used to call the gene expression level according to the ensembl gene annotation (release 66). DEXseq software^22^ (release v1.4.0) was then used to identify differential exons usage and significant over and under-expressed genes were selected by using Benjamini & Hochberg adjusted *P* value (FDR ≤0.1).

### Computer simulation of action potential durations and ECG

Two mathematical models, O'Hara-Rudy (ORd) dynamic model^48^,^49^ of ventricular myocytes and rabbit atrio-ventricular node model^50^, were employed to simulate action potential of cardiac cells and ECG. For the simulation, parameters of the activation gates for control hNav1.5, an equimolar mix of hNav1.5 and hNav1.5e, and hNav1.5e were estimated to match the experimental data obtained with Xenopus oocytes. Na currents and curves of voltage dependence of control hNav1.5, an equimolar mix of hNav1.5 and hNav1.5e, and hNav1.5e were reconstructed using these estimated parameters (Supplementary Fig. 5A–D). The parameters for the inactivation gate were unchanged. To get ECG similar to the left precordial ECG, a unipolar recording electrode was located 2.6 cm far from the epicardial border of the ventricular wedge preparation model (Supplementary Fig. 5E). The model consists of endocardial (Endo), midmyocardial (M) and epicardial (Epi) layers of thicknesses 0.12, 0.65 and 0.24 cm, respectively. The membrane kinetics and the transmural heterogeneity were based on the ORd model^48^ with twice sodium channel conductance to reproduce physiological ranges of both ventricular conduction velocity (∼60 cm s^−1^ along the fibre) and tissue excitability. The pacing stimuli of 2 ms and twice diastolic threshold were applied transmembranously to the part of endocardial border at 1 Hz to achieve steady state. The action potentials of Endo, M and Epi are recorded at the centre of each myocardial layer. Temperature employed in the simulation was 37 °C. Ionic concentrations employed in the simulation were 96 mM NaCl, 2 mM KCl, 1.8 mM CaCl2, 1 mM MgCl2, 10 mM HEPES/KOH and pH 7.4. Other model parameters and the numerical approach have been described elsewhere^49^. One-dimensional mathematical model^50^ of atrio-ventricular node was employed to simulate the conduction between atrium to ventricle. Although this model was based on the experimental data in rabbit heart, this is, in our knowledge, the only model for AV conduction. The simulation parameters for the equimolar mix of hNav1.5 and hNav1.5e and hNav1.5e were set to reproduce the shift inactivation and the decrease of peak Na current observed in oocytes. The multicellular model consisted of atrium and AV node, and action potentials of atrium and AV node cells were simulated with the stimulus intervals of 350 ms.

### Construction and analysis of the SCN5A minigenes

Exons 5, 6A, 6B and 7 were PCR amplified from human DNA (Clontech) with FWD primer: 5′- aaaagctagcgtacaccttcaccgccatttacacc -3′ and REV primer: 5′- aaaagcggccgcggatcactgaggtaaaggtccagg -3′, and inserted between the NheI and NotI restriction sites of pCDNA3.1+ (Invitrogen). Mutations in *SCN5A* intron 5 were introduced by primer-directed PCR mutagenesis. C2C12 cells plated in 6-well plates were co-transfected with Lipofectamine 2000 (Invitrogen) according to the manufacturer's instructions with SCN5A minigene and DMPKS DT960, V5-CUGBP1, V5-MBNL1 40 kDa (NM_207292) and siRNA directed against MBNL1 (5′- CACGGAAUGUAAAUUUGCAdTdT -3′; Eurogentec) or against CUGBP1 (NM_198700; NM_006560; NM_001025596; NM_001172640; NM_001172639; Thermo Scientific) in DMEM medium containing 1 g per l glucose and 2% horse serum (37 °C, 5% CO_2_) during 24 h. Total RNAs were extracted using Tri Reagent (MRC) and subjected to reverse transcription using Transcriptor Reverse Transcriptase kit (Roche). PCR were performed using Taq polymerase (Roche), one denaturation step at 94 °C for 2 min, 26 cycles of amplification 94 °C for 1 min, 58 °C for 30 s., 72 °C for 45 s and a final step at 72 °C for 7 min using the forward primer (5′- cttctgcctgcacgcgttcac -3′) and the reverse (5′- acgggccctctagactcg -3′) specific to pCDNA3.1- SCN5A minigene. The PCR products were digested by *BstB*I enzyme. PCR products of mRNAs including exon 6A and 6B are of 116+71 bp and 187 bp, respectively. PCR products were precipitated, analysed by electrophoresis on 6,5% polyacrylamide gel, BET labelled and quantified using Typhoon scanner.

### Electrophysiological measurements

Ovarian lobes were obtained from *Xenopus laevis* under anaesthesia (0.4% 3-aminobenzoic acid ethyl ester) and transferred to Ca^2+^-free Barth medium (84 mM NaCl, 1 mM, 2.4 mM NaHCO3, 0.82 mM MgSO4 and 7.5 mM Tris/HCl pH 7.4). Oocytes in stages V and VI were incubated in Ca^2+^-free Barth medium containing 1.2 mg ml^−1^ collagenase for 60 min. After washing repeatedly with Barth medium, the oocytes were isolated and defolliculated mechanically. Plasmids containing cDNAs of hNav1.5 or hNav1.5e were linearized using the *Not*I restriction enzyme and *in vitro* transcribed using the T7 RNA polymerase. Concentrations of the cRNA variants were adjusted to ∼0.002 μg μl^−1^ using the gel documentation system from Herolab (Wiesloch, Germany) and a reference RNA sample (0.24–9.5 kb RNA ladder, Invitrogen Life Technologies), before injecting ∼50–80 nl cRNA per oocyte. After 3 days incubation at 18 °C in Barth medium, the peak current amplitude of the whole-cell Na^+^ current was between 0.5 and 7.5 μA, depending on the quality of the oocyte batch. Cells producing currents larger than 6 μA were not selected for the evaluation of the channel kinetics. Measurements were performed in totally 11 different batches of oocytes. Whole-cell Na^+^ currents were recorded with the two-microelectrode voltage-clamp technique using the TEC-05-S amplifier (npi electronic GmbH, Tamm, Germany) and the following bath solution (96 mM NaCl, 2 mM KCl, 1.8 mM CaCl2, 1 mM MgCl2, 10 mM HEPES/KOH and pH 7.4). Currents were elicited by test potentials from −80 to 20 mV in 5 or 10 mV increments at a pulsing frequency of 1.0 Hz (holding potential −120 mV). Steady-state activation (m∞) was evaluated by fitting the Boltzmann equation m∞={1+exp[−(*V*−Vm) per *s*]}−1 to the normalized conductance as function of voltage, where *V* is the test potential, Vm the mid-activation potential, and *s* the slope factor in mV. Recording and analysis of the data was performed on a personal computer with the ISO3 software (MFK, Niedernhausen, Germany). The sampling rate was 50 kHz. Student´s *t*-test was used to test for statistical significance. Statistical significance was assumed for *P*<0.05.

### *In vivo* gene transfer

All mouse procedures were done according to protocol approved by the Committee on Animal Resources at the Centre d'Exploration Fonctionnelle of UPMC animal facility and under appropriate biological containment. AAV2/9 vectors expressing *Scn5a* antisense sequences driven by an optimized U7-snRNA (U7-AS) construct described previously^73^ were produced by co-transfection in HEK293 cells of pAAV2(U7-AS), pAD-DELTA-F16 encoding adenovirus helper functions and p5E18-VD2/9. Vector particles were purified on iodixanol gradients from cell lysates obtained 72 h after transfection, and titres were measured by RT-qPCR. The vector preparation used in this study had a titer of 1 × 10EXP13 vector genomes (vg) ml^–1^. U7-AS constructs that contain antisense sequences to target intron 6/exon 6B border or exon 6B of *Scn5A* were cloned in the same AAV backbone. 2 days old C57BL/6 mice were injected in the temporal vein for systemic delivery with 50 μl of either with saline, empty AAV2/9 or AAV2/9-U7-AS (1 × 10^12^ vector genomes ml^−1^). Six months after injections, mice were killed, hearts were collected and snap-frozen in liquid nitrogen-cooled isopentane and stored at −80 °C.

### ECG recordings and echocardiographic examination

Mice were anaesthetized with isoflurane and surface ECG was recorded using 23-gauge, subcutaneous needle electrodes (Gould Instrument Systems), affixed to each limb. ECG was continuously monitored for 3 min (software IOX 2, EMKA Technologies, Paris France). Heart rate, PR and QRS duration were measured from recording traces using ECG auto V.1.5.7 software (Emka Technologies). Non-invasive measurements of left ventricular dimensions were evaluated under isoflurane anaesthesia, using echocardiography-Doppler (General Electric Medical systems Co, Vivid 7 Dimension/Vivid 7 PRO) with a probe emitting ultrasounds with 9–14 MHz frequency. The two-dimensionally guided Time Motion mode recording of the left ventricle provided the following measurements: diastolic and systolic septal and posterior wall thicknesses, internal end-diastolic and end-systolic diameters, ejection fraction, fractional shortening of left ventricular diameter. Each set of measurements was obtained from a same cardiac cycle. At least three sets of measures were obtained from three different cardiac cycles.

### Recombinant protein production and purification

*Escherichia coli* BL21(RIL) pRARE competent cells (Invitrogen) were transformed with peGEX-MBNL1Δ^101^, which is a C-terminal truncated form of MBNL1 with higher solubility but identical affinity than MBNL1 full-length, (gift from Prof. C. Branlant), grown at 37 °C in 400 ml of LB medium supplemented with Kanamycin until OD600=0.5, 0.5 mM IPTG was added and the culture was further incubated 4 h at 30 °C. Harvested cells were sonicated in 50 mM Tris-Cl pH 7.5, 300 mM NaCl, 5% glycerol, 1 mM DTT, 5 mM EDTA, centrifuged 20 min at 20,000*g* and recombinant GST-MBNL1^Δ101^, protein was purified using GST-Kits (Novagen) and store in 20% glycerol.

### RNA transcription and gel-shift assays

Templates for transcription were obtained by PCR with pCDNA3.1-SCN5A or pCDNA3.1-SCN5A mutant minigenes and the primers (FWD that include a T7 promoter sequence: 5′- TAATACGACTCACTATAGGGCCATGGAACTGGCTGGACTTT -3′ and REV: 5′- AGAAAGAGAGGGGTGGTCG -3′). Transcription reaction was performed using T7 transcription kit (Ambion) in presence of 1 μl of αP32-CTP (10μCi, 800 Ci per mmole, Perkin Elmer), analysed on 8% denaturing polyacrylamide and quantified with LS-6500 counter (Beckman). After transcription, 1 unit of DNase I (Invitrogen, Carlsbad, CA) was added, and the sample was incubated for additional 30 min at 37 °C. Transcribed RNAs were then purified by micro Bio-Spin 6 chromatography columns (Bio-rad) according to the manufacturer's instructions. The sizes of RNAs were checked by gel electrophoresis on a denaturing 6% polyacrylamide gel. For gel shift, 1 μl of labelled RNA was annealed in binding buffer (BB, 50 mM Tris-HCl (pH 7.0), 75.0 mM NaCl, 37.5 mM KCl, 5.25 mM DTT, 0.1 mg ml^−1^ BSA, 0.1 mg ml^−1^ *E. Coli* tRNA) at 90 °C for 5 min. and allowed to cool to room temperature by placing on a bench top. After cooling, MgCl2 and RNAsin were added to a final concentration of 0.75 mM and 0.4 U μl^−1^ respectively. GST-MBNL1-Δ101 protein was then added to the above solution and the mixture was incubated at 25 °C for 20 min. The solution mixture was loaded onto a non-denaturing 6.0% (w/v) polyacrylamide gel (acrylamide/bisacrylamide, 40:1, w/w) containing 0.5 × TBE (1X TBE is 90 mM Tris-base, 89 mM Boric acid and 2 mM EDTA (pH 8.0)), which had been pre-electrophoresed at 110 V for 20 min. at 4 °C. The gel was electrophoresed at 110 V at 4 °C for 3 h. The gel was then dried and exposed to a phosphorimager screen and imaged using a Typhoon 9410 variable mode imager. The data were fit to the following equation: *y*=min+((max−min))/(1+(x/IC50)-HillSlope)), where *y* is the percentage of RNA bound to MBNL1, *x* is the concentration of MBNL1, min and max are the minimum and maximum percentage of RNA bound to MBNL1 (0–100%) and IC50 is the concentration where 50% of maximum binding is achieved.

### RT–PCR analyzes

Total RNA was isolated by TriReagent (Molecular Research Center). A total of 1 μg of total RNA isolated from heart samples of control or DM individual was subjected to reverse transcription using random hexanucleotide and the Transcriptor High Fidelity cDNA synthesis kit (Roche Diagnostics). PCR was performed with Taq polymerase (Roche), using the following protocol: one denaturation step at 94 °C for 2 min, 30 cycles of amplification 94 °C for 1 min, 60 °C for 1 min, 72 °C for 2 min and a final step at 72 °C for 5 min using primers described in the Supplementary Table 4. The PCR products were precipitated, analysed by electrophoresis on a 6.5% polyacrylamide gel, stained with ethidium bromide and quantified using a PhosphorImager Typhoon scanner (GE Healthcare) and the ImageQuaNT software. Concerning analysis of alternative splicing of human *SCN5A*, the forward primer was located within *SCN5A* exon 5 (FWD: 5′- CTTCTGCCTGCACGCGTTCAC -3′) and the reverse primer within *SCN5A* exon 7 (REV: 5′- CAGAAGACTGTGAGGACCATC -3′). Since *SCN5A* exons 6A or 6B are of similar size (92 nts), PCR products were digested by *BstB*I enzyme before loading on 6.5% polyacrylamide gel, resulting in PCR products of 240 bp (exon 6B) and a doublet of 115 bp and 125 bp (exon 6A digested by *BstB*I).

### Quantitative real-time PCR analyzes

Total RNA was isolated by TriReagent (Molecular Research Center). cDNAs were generated using using the Transcriptor High Fidelity cDNA synthesis kit (Roche Diagnostics). Real-time qPCR were realized using the LightCycler 480 SYBR Green I Master (Roche) in a Lightcycler 480 (Roche) with 15 min at 94 °C followed by 50 cycles of 15 s at 94 °C, 20 s at 58 °C and 20 s at 72 °C using primers described in the Supplementary Table 5. *Rplpo* mRNA was used as standard and data were analysed using the Lightcycler 480 analysis software (2ΔCt method).

## Additional information

**Accession codes:** ExonArray and RNA-Seq data have been deposited in Gene Expression Omnibus (GEO) data base under accession codes GSE67067 and GSE67812, respectively.

**How to cite this article:** Freyermuth, F. *et al*. Splicing misregulation of *SCN5A* contributes to cardiac-conduction delay and heart arrhythmia in myotonic dystrophy. *Nat. Commun.* 7:11067 doi: 10.1038/ncomms11067 (2016).

## Supplementary Material

Supplementary Figures and Tables Supplementary Figures 1-5 and Supplementary Tables 1-5

Supplementary Data Set 1 MISO analysis

Supplementary Data Set 2 DEXSeq analysis
