# Supplementary material for: Splicing misregulation of SCN5A contributes to cardiac-conduction delay and heart arrhythmia in myotonic dystrophy
Source: Nat Commun. 2016 Apr 11;7:11067. doi: 10.1038/ncomms11067 (PMC4831019; doi:10.1038/ncomms11067)
Supplement: Supplementary Figures and Tables — Supplementary Figures 1-5 and Supplementary Tables 1-5 [file ncomms11067-s1.pdf]

Supplementary Figure 1

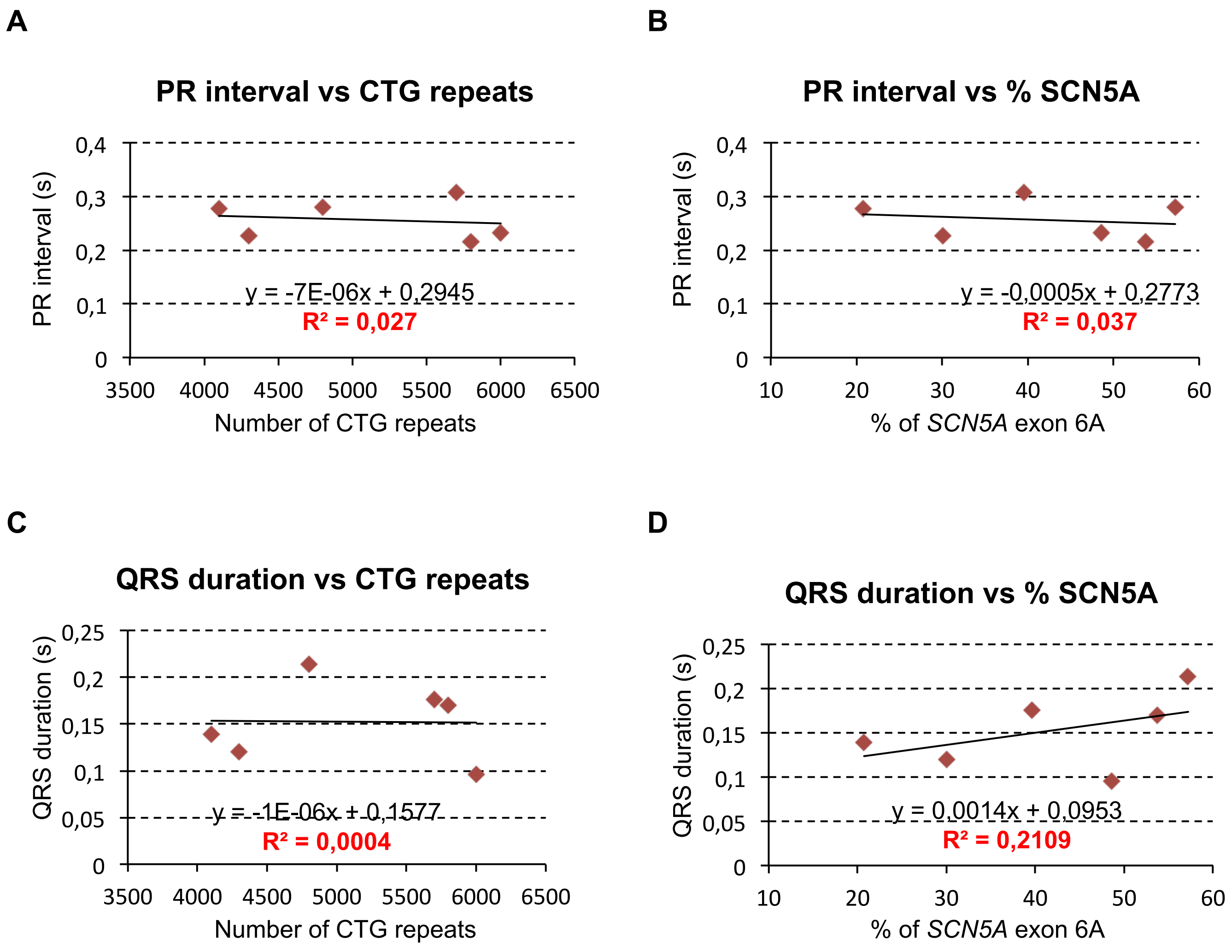

Supplementary Figure 1.
Absence of correlation between alteration of the PR interval and the number of CTG repeats in heart (**A**) or the splicing alteration of *SCN5A* (**B**) in six adult DM1 patients free of pacemaker. Similary, there is no correlation between the alteration of the QRS duration and the number of CTG repeats in heart (**C**) and only a very limited (R^2 of 0.2) correlation with the splicing alteration of *SCN5A* (**D**) in adult DM1 patients free of pacemaker (n=6).

Supplementary Figure 2

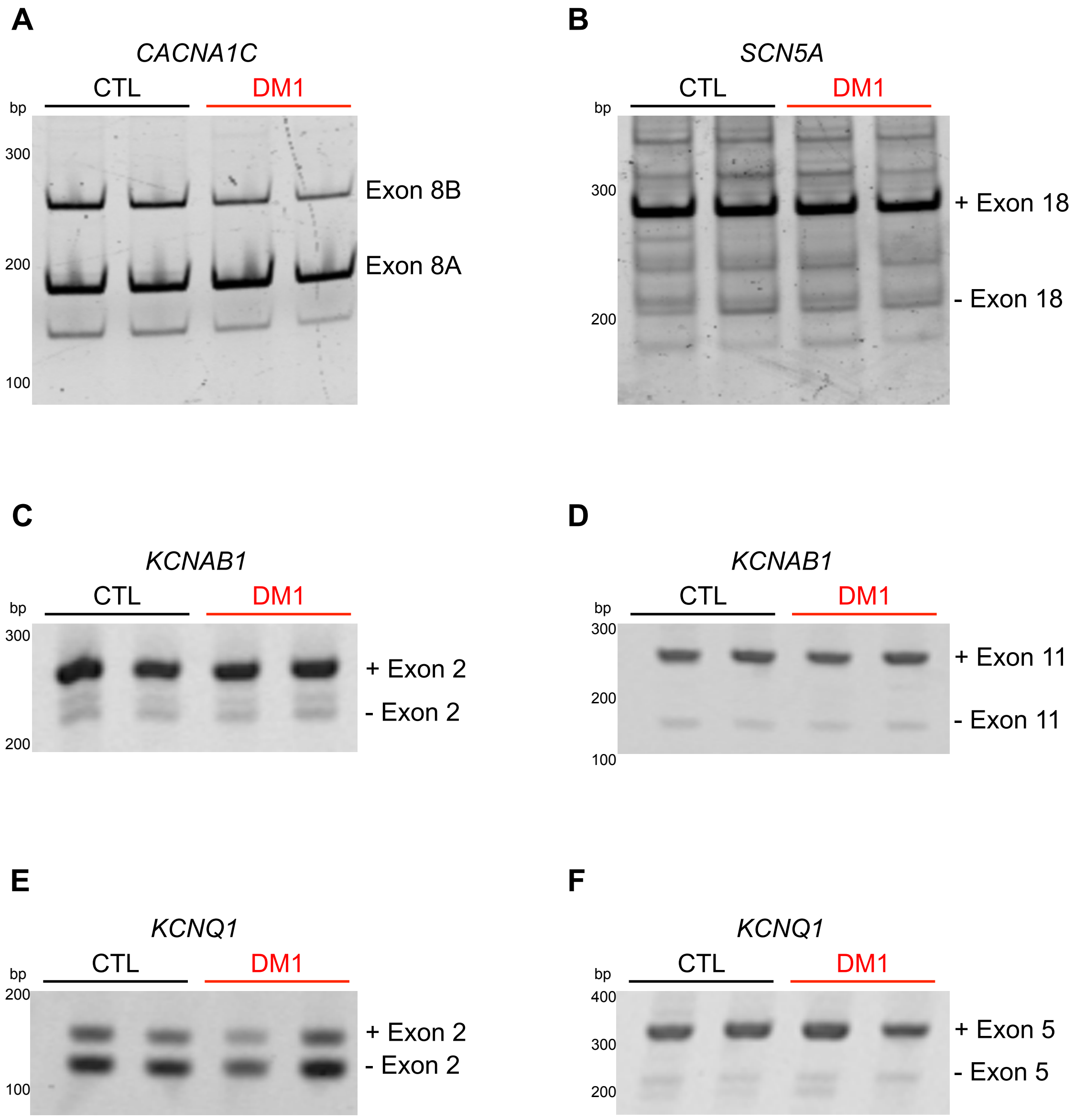

Supplementary Figure 2.
Control RT-PCR showing no mis-regulation of the alternative splicing of *CACNA1C* mutually exclusive exons 8A/8B, *SCN5A* exon 18, *KCNAB1* exons 2 and 11 and *KCNQ1* exons 2 and 5 in human heart samples of adult DM1 patients (DM1, red) compared to normal adult individuals (CTL, black). Molecular size markers are in base pairs (bp).

Supplementary Figure 3

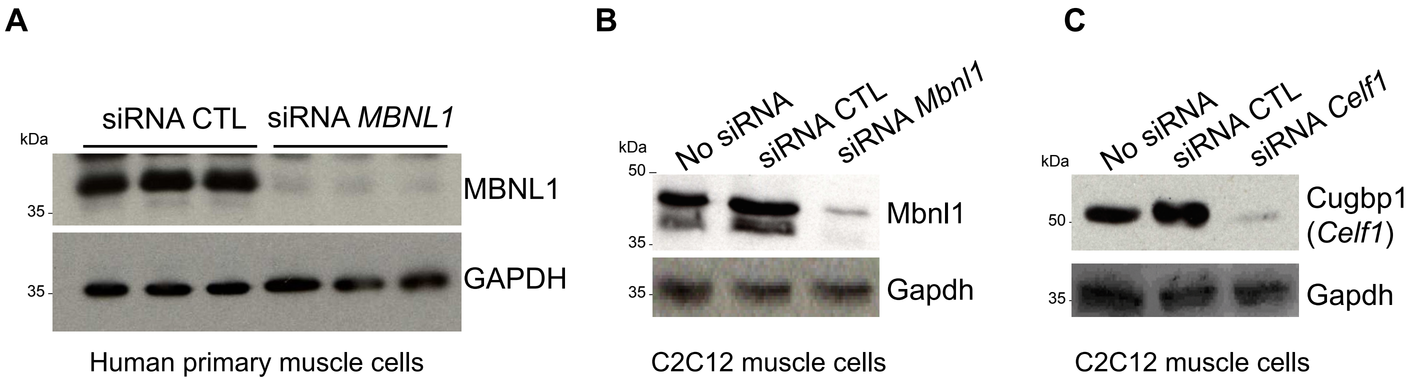

Supplementary Figure 3.
Western blotting demonstrating efficient decreased expression of MBNL1 (**A** and **B**) or of Cugbp1(**C**) upon transfection of siRNA in human primary muscle cell cultures (**A**) or in mouse C2C12 cells (**B** and **C**). Molecular weight markers are in kilodalton (kDa). One representative immunoblot of three independent experiments is shown.

Supplementary Figure 4

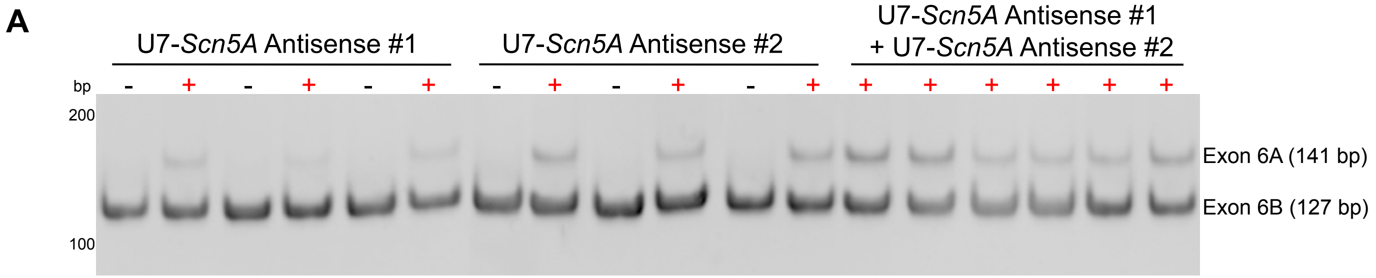

B

	4 months			6 months		
	**CTL** (n=17)	**U7-AS^{Scn5a}** (n=25)	**Student t-test p value**	**CTL** (n=8)	**U7-AS^{Scn5a}** (n=15)	**Student t-test p value**
**P duration** (ms)	20,47±0,7	21,04±1,1	ns	19,17 ± 1	21,02 ± 0,9	ns
**PR** (ms)	34,16±0,8	39,28±0,9	<0,001	34,78 ± 1,3	40,46 ± 1,4	<0,05
**QRS** (ms)	16,5±0.6	18±0,5	ns (0.058)	15,93 ± 0,8	16.94 ± 0,8	ns
**QTc** (ms)	49,28±1,1	51,7±0,9	ns	51,78 ± 3,1	50,08 ± 1,7	ns
**RR** (ms)	122,4±2,7	138,4±3,7	<0,05	136,5 ± 8,7	143,6 ±4,7	ns
**HR** (bpm)	479,4±11,58	450,4±10,93	ns	447,4±22,22	422,4±14,95	ns

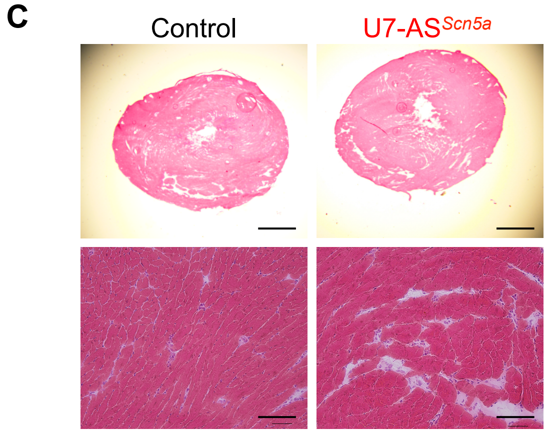

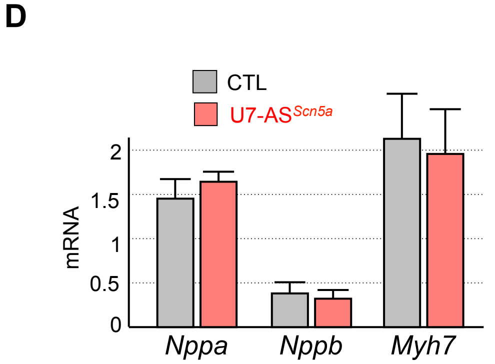

Supplementary Figure 4.
(**A**) RT-PCR analysis of the alternative splicing of endogenous *Scn5a* mRNA from heart samples of mice injected with AAV2/9 expressing either one or two *Scn5a* antisense oligonucleotides (noted +) compared to control injected mice (noted -). PCR were performed with oligonucleotides *Scn5a* Fwd: CTGGTCAAGATTCTAGCTCGAGG, 6a Rev: TCGAAGAGCCGACAAATTGC and 6b Rev: CATTGCCCAGGTCCACAAATTCAG. Molecular size marker is in base pairs (bp)(**B**) Electrocardiography recording in control or AAV-U7-AS^{Scn5a} injected mice. (**C**) Upper panel, normal heart structure and H&E staining in control or AAV-U7-AS^{Scn5a} injected mice. Scale bars, 1 mm. Lower panel, magnification reveals evidence of mild fibrosis in AAV-U7-AS^{Scn5a} injected mice. Scale bars, 100 µm. Representative image of 6 analyzed heart samples. (**D**) Real-time RT-qPCR quantification of the expression of control *Nppa*, *Nppb* and *Myh7* mRNAs in heart of control (n=6) or AAV-U7-AS^{Scn5a} injected (n=6) mice. Bars indicate s.e.m.

Supplementary Figure 5

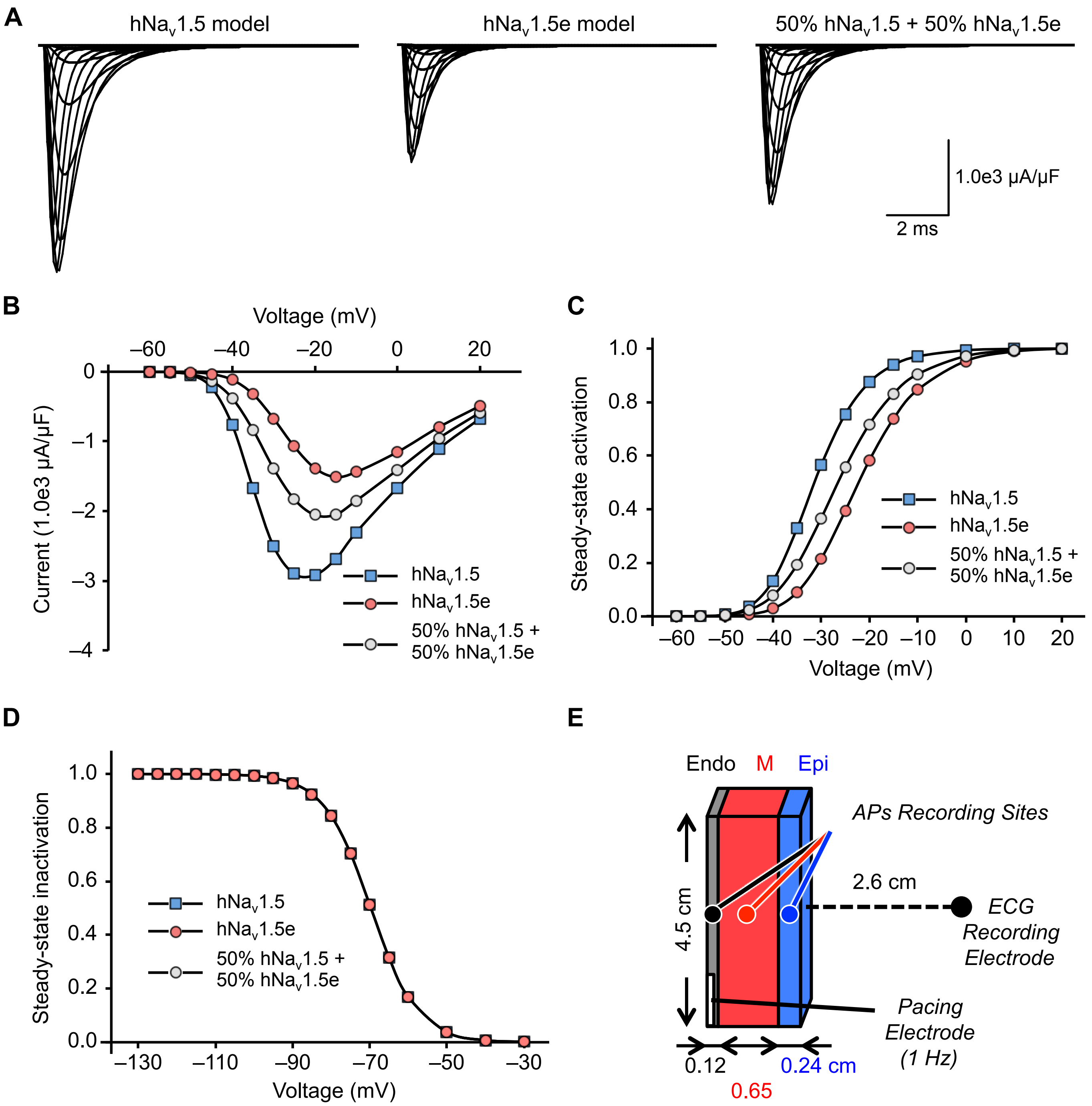

Supplementary Figure 5.
Reconstructed Na currents (**A**) and voltage-dependent curves for activation (**B,C**) and inactivation (**D**) using parameters for each Na channels estimated from our electrophysiological study. Supplemental Figure 4 A, B, C and D correspond to the Figure 5 A, C, D and F, respectively. (**E**) Schematic illustration of the ventricular wedge preparation model. The model parameters for transmural heterogeneity (Endo, M and Epi) were based on O'Hara T et al (2011). Note that the current amplitude of the simulated Na+ current in the present study was higher (up to 10 fold) than usually expected. However, such a high current density was derived from the balance of ion channel currents included in the original ORd model, and thus we believe that the current amplitude of the simulated Na+ current was in the physiological range of our model system.

Supplementary Table 1

Symbol	Name	Regulation	FC	P-value	AS Event	Exon position	Nb probes analysed	Human Ensembl id	Chr Position
ABLIM1	actin binding LIM protein 1 [Source:HGNC Symbol;Acc:78]	down	4,3	3,5E-02	exon	e14:282881-282964	4	ENSG00000099204	10(-1):116190870-116528019
MYOM1	myomesin 1, 185kDa [Source:HGNC Symbol;Acc:7613]	up	4,1	2,8E-04	exon	e18:90590-90877	8	ENSG00000101605	18(-1):3066805-3220106
SORBS2	sorbin and SH3 domain containing 2 [Source:HGNC Symbol;Acc:24098]	up	3,7	2,0E-02	exon	e8:271871-271963	4	ENSG00000154556	4(-1):186506598-186877870
LDB3	LIM domain binding 3 [Source:HGNC Symbol;Acc:15710]	down	3,3	8,3E-02	exon	e8:20278-20481	4	ENSG00000122367	10(1):88426549-88495825
NCAM1	neural cell adhesion molecule 1 [Source:HGNC Symbol;Acc:7656]	down	2,9	5,1E-02	exon	e16:109878-109923	3	ENSG00000149294	11(1):113003650-113149148
MBNL2	muscleblind-like 2 (Drosophila) [Source:HGNC Symbol;Acc:16746]	up	2,7	9,2E-02	exon	e8:214961-215014	3	ENSG00000139793	13(1):97794090-98046374
**SCN5A**	**sodium channel, voltage-gated, type V, alpha subunit [Source:HGNC Symbol;Acc:10593]**	**down**	**2,5**	**1,4E-02**	**exon**	**e8:35840-35931**	**3**	**ENSG00000183873**	**3(-1):38589548-38691164**
CAMK2B	calcium/calmodulin-dependent protein kinase II beta [Source:HGNC Symbol;Acc:1461]	up	2,4	4,2E-03	exon	e17:94578-94626	4	ENSG00000058404	7(-1):44256749-44365230
PPFIBP1	PTPRF interacting protein, binding protein 1 (liprin beta 1) [Source:HGNC Symbol;Acc:9249]	down	2,3	5,9E-02	exon	e20:152953-152985	3	ENSG00000110841	12(1):27677045-27848496
MBNL1	muscleblind-like [Source:HGNC Symbol;Acc:6923]	up	2,3	3,8E-02	exon	e10:178665-178718	3	ENSG00000152601	3(1):151985829-152183568
PRSS50	protease, serine, 50 [Source:HGNC Symbol;Acc:17910]	down	2,3	6,0E-05	exon	e2:702-1469	18	ENSG00000206549	3(-1):46753605-46854064
SGIP1	SH3-domain GRB2-like (endophilin) interacting protein 1 [Source:HGNC Symbol;Acc:25412]	down	2,2	1,1E-02	exon	e16:148487-148987	10	ENSG00000118473	1(1):66999066-67213982
TMEM49	transmembrane protein 49 [Source:HGNC Symbol;Acc:29559]	up	2,2	3,0E-02	exon	e11:130794-130896	4	ENSG00000062716	17(1):57784863-57918453
CPXM2	carboxypeptidase X (M14 family), member 2 [Source:HGNC Symbol;Acc:26977]	down	2,1	9,1E-02	exon	e18:226713-226822	3	ENSG00000121898	10(-1):125465729-125699785
GOLGA4	golgin A4 [Source:HGNC Symbol;Acc:4427]	up	2,1	1,8E-02	exon	e28:118053-119031	9	ENSG00000144674	3(1):37284682-37408370
PAM	peptidylglycine alpha-amidating monooxygenase [Source:HGNC Symbol;Acc:8596]	up	2,1	4,9E-02	exon	e15:121696-121749	4	ENSG00000145730	5(1):102201527-102366792
CAMK2B	calcium/calmodulin-dependent protein kinase II beta [Source:HGNC Symbol;Acc:1461]	up	2,1	1,0E-02	exon	e16:92766-92810	3	ENSG00000058404	7(-1):44256749-44365230
TNNT2	troponin T type 2 (cardiac) [Source:HGNC Symbol;Acc:11949]	up	2,1	7,4E-02	exon	e3:5608-5736	4	ENSG00000118194	1(-1):201328142-201346890
TPM2	tropomyosin 2 (beta) [Source:HGNC Symbol;Acc:12011]	down	2,0	1,8E-02	exon	e6:4919-4994	4	ENSG00000198467	9(-1):35681990-35690054
PFKFB2	6-phosphofructo-2-kinase/fructose-2,6-biphosphatase 2 [Source:HGNC Symbol;Acc:8873]	up	2,0	4,6E-02	exon	e6:18660-18768	4	ENSG00000123836	1(1):207215508-207254368
INMT	indolethylamine N-methyltransferase [Source:HGNC Symbol;Acc:6069]	up	2,0	5,6E-02	exon	e28:224579-224659	4	ENSG00000241644	7(1):30737601-30963427
AKAP13	A kinase (PRKA) anchor protein 13 [Source:HGNC Symbol;Acc:371]	down	2,0	9,6E-02	exon	e15:281803-281868	4	ENSG00000170776	15(1):85923817-86292586
PDLIM5	PDZ and LIM domain 5 [Source:HGNC Symbol;Acc:17468]	down	2,0	5,9E-02	exon	e11:135106-136335	12	ENSG00000163110	4(1):95373035-95589377
SH3RF2	SH3 domain containing ring finger 2 [Source:HGNC Symbol;Acc:26299]	down	2,0	2,1E-02	exon	e12:141898-145211	5	ENSG00000156463	5(1):145316126-145461336

Supplementary Table 1.
Alternative splicing changes predicted by paired comparisons analysis of GeneChip Human Exon 1.0 ST array of 3 controls and 3 DM1 heart samples. Only the most significant (FC >2, Student t-test p-value <0.01) misregulations are presented.

Supplementary Table 2

Channel	Peak current at -10 mV (μA)	Steady-state activation S (mV)	Steady-state activation V_m (mV)	N
$hNa_v1.5$ (Exon 6B)	3.67 ± 0.40	3.7 ± 0.1	-33.6 ± 0.7	25
$hNa_v1.5e$ (Exon 6A)	2.03 ± 0.21	4.2 ± 0.1	-26.6 ± 0.7	27
$hNa_v1.5$ + $hNa_v1.5e$ (Exon 6A + exon 6B)	2.58 ± 0.29	4.1 ± 0.1	-29.8 ± 0.6	25

Supplementary Table 2.
Electrophysiological properties of hNav1.5e and hNav1.5 channels, containing respectively SCN5A fetal exon 6A or adult exon 6B, expressed in *Xenopus* oocytes.

Supplemental Table 3

	4 months		6 months	
	**CTL** (n=17)	**U7-AS^{Scn5a}** (n=24)	**CTL** (n=8)	**U7-AS^{Scn5a}** (n=15)
**Heart rate (bpm)**	568.3 ± 29.5	562.2 ± 38.2	568.1 ± 30.5	569.18 ± 42.1
**IVSd (cm)**	0.06 ± 0.006	0.06 ± 0.005	0.06 ± 0.007	0.06 ± 0.004
**LVEDd (cm)**	0.35 ± 0.03	0.36 ± 0.03	0.35 ± 0.02	0.37 ± 0.03
**PWd (cm)**	0.082 ± 0.1	0.079 ± 0.008	0.078 ± 0.01	0.081 ± 0.01
**IVSs (cm)**	0.104 ± 0.008	0.098 ± 0.006	0.101 ± 0.009	0.100 ± 0.008
**LVEDs (cm)**	0.191 ± 0.02	0.204 ± 0.02	0.193 ± 0.01	0.208 ± 0.03
**PWs (cm)**	0.116 ± 0.01	0.115 ± 0.01	0.11 ± 0.01	0.12 ± 0.01
**LVEF Teichholtz (%)**	82.3 ± 1	80.1 ± 3.3	81.2 ± 1	80.8 ± 3.8
**Shortening fraction (%)**	44.9 ± 1.1	42.9 ± 3.1	43.8 ± 1	43.6 ± 3.1
**RWT**	0.42 ± 0.04	0.39 ± 0.04	0.40 ± 0.04	0.39 ± 0.04
**Cardiac output (l/min)**	0.052 ± 0.01	0.052 ± 0.01	0.05 ± 0.01	0.057 ± 0.01

Supplementary Table 3.
Echography properties of mouse heart injected with AAV2/9 expressing *Scn5a* antisense oligonucleotides compared to control injected mice. IVSd: intraventricular septum thickness. LVID: left ventricular end diameter. PW: posterior wall. d: diastolic wave. s: systolic wave. LVEF: left ventricular ejection fraction. RWT: relative wall thickness. No significant (student t-test) alterations were observed.

Supplemental Table 4

**HUMAN**	
ABLIM1 FWD	GTTTGGCATCCCGACTGTAAG
ABLIM1 REV	GAGCCAGGAATACTGGAGCCTGG
ADD3 FWD	CCACCAGCTCCTCCTAACC
ADD3 REV	TACCATGACAGGCACTTCCA
ANK3 FWD	CCCTGTGGTCGTCTGTCTTT
ANK3 REV	CCAGGCTCAGTCAAGTAGCTG
ARHGEF10L FWD	CGAGAGCTACAGCGAGGACT
ARHGEF10L REV	GTGCAGGAAGGAGACTTTGC
CACNA1C FWD	TCATCTTTGGATCCTTTTTCG
CACNA1C REV	CTTGGCCTTCTCCCTCTCTT
CLASP1 FWD	TGCCAAATCCAAAGTCTCCT
CLASP1 REV	CCCCGGTTATCAGGTGTAGA
CLTB FWD	AAGGTCACGGAACAGGAATG
CLTB REV	CTGCTCTTGGGGTTGAAGTC
CRTC2 FWD	CATTATGGGACACCGTACCC
CRTC2 REV	CCTAAAAATCCAGGCCCTTC
CAMK2B FWD	TGACAGTGCCAATACCACCA
CAMK2B REV	GCCTCAAAGTCACCGTTGTT
EPN2 FWD	AGCAACCAGATCACCTTTGG
EPN2 REV	CTGCAGCTGAAGCTCCTCTT
GOLGA4 FWD	AGAGAAGATGCTCGGCTGAT
GOLGA4 REV	TTCTCAAAGAGTAGCAAGTGTGG
MBNL2 FWD	TACCAGCGCGGTCTTTAAC
MBNL2 REV	TTATTCTCAATGCAGATTCTTGG
MXRA7 FWD	GGCTTCTCCTTCAAATACAGC
MXRA7 REV	AAGATGCCAAAAGGAAAAGC
MYH11 FWD	CACCAAGAAGGTCATTCAGTACC
MYH11 REV	CTCCAGAATCGGGTTTGCT
NUMA1 FWD	GGAGGTGATGACTGCCAAGT
NUMA1 REV	CTTCTGCTGCTGCACCTTG
SCN5A FWD	CTTCTGCCTGCACGCGTTCAC
SCN5A REV	CAGAAGACTGTGAGGACCATC
TECR FWD	CTGTAGGGAGCCTGTGCTGT
TECR REV	CCTTGTCTTTGCGTCCAGA
TPM2 FWD	GAAGCTGGTGATCCTGGAAG
TPM2 REV	TCCTTCAGCTTCTCCTCCAA
UNC13B FWD	CTCTCATCAAGACCTTTGTGC
UNC13B REV	CCCCAGTACCAGGGTGTGTA
**MOUSE**	
Scn5a FWD	CTGGTCAAGATTCTAGCTCGAGG
Scn5a REV	AGTAAGGACCATCACATCGGCTAGC

Supplementary Table 4.
Sequence of the oligonucleotide used to determine alternative splicing alterations by RT-PCR in heart samples of DM1 individuals (upper lanes) or in hear samples of AAV injected mice (bottom lanes).

Supplemental Table 5

Rplp0 FWD	GAGGACCTCACTGAGATTCGG
Rplp0 REV	TTCTGAGCTGGCACAGTGAC
Scn5a FWD	CCCAGAAGCAGGATGAGAAG
Scn5a REV	GATCTGGCAGCTTTTTGGAG
Scn1b FWD	CTGCGTGGAGGTGGATTCCG
Scn1b REV	GGCTGGCTCTTCCATGAGGC
Gja1 FWD	ACGGCAAGGTGAAGATGAGA
Gja1 REV	GAGAGACACCAAGGACACCA
Nppa FWD	GGGGGTAGGATTGACAGGAT
Nppa REV	GCAGAATCGACTGCCTTTTC
Nppb FWD	GTCAGTCGTTTGGGCTGTAAC
Nppb REV	AGACCCAGGCAGAGTCAGAA
Myh7 FWD	GACCAGACCCCAGGCAAGGG
Myh7 REV	GCCAACTTTCCTGTTGCCCC
Col1a1 FWD	AGCGGAGAGTACTGGAT
Col1a1 REV	CTTCTTTTCCTTGGGGTT
Col3a1 FWD	GCCCACAGCCTTCTACAC
Col3a1 REV	CCAGGGTCACCATTTCTC
Tgfβ1 FWD	GCAGTGCCCGAACCCCCAT
Tgfβ1 REV	GGGTCAGCAGCCGGTTAC

Supplementary Table 5.
Sequence of the oligonucleotide used to determine mRNA expression alterations by RT-qPCR in heart samples of AAV injected mice.
